# Computational inference of chemokine-mediated roles for the vagus nerve in modulating intra- and inter-tissue inflammation

**DOI:** 10.3389/fsysb.2024.1266279

**Published:** 2024-02-15

**Authors:** Ashti M. Shah, Ruben Zamora, Derek Barclay, Jinling Yin, Fayten El-Dehaibi, Meghan Addorisio, Tea Tsaava, Aisling Tynan, Kevin Tracey, Sangeeta S. Chavan, Yoram Vodovotz

**Affiliations:** ^1^ Department of Surgery, University of Pittsburgh, Pittsburgh, PA, United States; ^2^ Center for Inflammation and Regeneration Modeling, McGowan Institute for Regenerative Medicine, University of Pittsburgh, Pittsburgh, PA, United States; ^3^ Institute of Bioelectronic Medicine, Feinstein Institutes for Medical Research, Northwell Health, Manhasset, NY, United States

**Keywords:** vagus nerve, vagotomy, inflammation, chemokines, systems biology

## Abstract

**Introduction:** The vagus nerve innervates multiple organs, but its role in regulating cross-tissue spread of inflammation is as yet unclear. We hypothesized that the vagus nerve may regulate cross-tissue inflammation via modulation of the putatively neurally regulated chemokine IP-10/CXCL10.

**Methods:** Rate-of-change analysis, dynamic network analysis, and dynamic hypergraphs were used to model intra- and inter-tissue trends, respectively, in inflammatory mediators from mice that underwent either vagotomy or sham surgery.

**Results:** This analysis suggested that vagotomy primarily disrupts the cross-tissue attenuation of inflammatory networks involving IP-10 as well as the chemokines MIG/CXCL9 and CCL2/MCP-1 along with the cytokines IFN-γ and IL-6. Computational analysis also suggested that the vagus-dependent rate of expression of IP-10 and MIG/CXCL9 in the spleen impacts the trajectory of chemokine expression in other tissues. Perturbation of this complex system with bacterial lipopolysaccharide (LPS) revealed a vagally regulated role for MIG in the heart. Further, LPS-stimulated expression of IP-10 was inferred to be vagus-independent across all tissues examined while reducing connectivity to IL-6 and MCP-1, a hypothesis supported by Boolean network modeling.

**Discussion:** Together, these studies define novel spatiotemporal dimensions of vagus-regulated acute inflammation.

## 1 Introduction

The vagus nerve is a parasympathetic nerve that comprises of 80% afferent fibers and 20% efferent fibers, innervating viscera throughout the body and acting as a central modulator of the stress response (Zagon, 2001; Bonaz et al., 2016). Signals transmitted in the vagus nerve play a key role in regulating inflammation, tonically regulating the key pro-inflammatory cytokines interleukin (IL)-6 and tumor necrosis factor-α (TNF) along with other pro-inflammatory chemokines, cytokines released by innate and adaptive immune cells via the inflammatory reflex (Tracey, 2002; Pavlov and Tracey, 2012). The efferent arm of the reflex results in the release of acetylcholine (Ach), which acts to suppress the local inflammatory response (Borovikova et al., 2000; Pavlov and Tracey, 2012). Given the anti-inflammatory effects of the vagus nerve, there have been extensive efforts to harness, leverage, and accentuate the natural anti-inflammatory properties of this pathway, and vagus nerve stimulation is approved by the U.S. Food and Drug Administration for the treatment of drug-resistant epilepsy and depression (Johnson and Wilson, 2018). Ongoing investigation into the use of vagal nerve stimulation for the treatment of rheumatoid arthritis, sepsis, fibromyalgia, Crohn’s disease, and other inflammatory diseases offers promising potential for the treatment of debilitating chronic inflammatory diseases (Koopman et al., 2016; Johnson and Wilson, 2018; Caravaca et al., 2019).

The inflammatory response is a prototypical complex system, a dynamic network regulated by thresholds and feedback loops requiring analysis using both data-driven and mechanistic computational modeling (Vodovotz, 2023). While inflammation in various syndromes such as critical illness elaborates across multiple tissues and organs and likely impacts organ failure (Bone, 1996), how this occurs is a matter of debate (Deutschman and Tracey, 2014). We have hypothesized that knowledge regarding the spread or attenuation of inflammation across tissues may help clarify how inflammation impacts organ pathophysiology, and have utilized data-driven computational modeling in the context of experimental murine endotoxemia to define the spatiotemporal spread of inflammation across multiple organs (Zamora et al., 2018; Zamora et al., 2021). The role of the vagus nerve in the dynamic evolution of intra- and inter-tissue inflammation is as yet unknown, but in the context of critical illness that ensues following severe traumatic injury, network modeling studies using data on systemic inflammatory mediators in trauma patients with an intact spinal cord vs spinal cord injury patients have pointed to the chemokine inducible protein of 10 kDa (IP-10/CXCL10) as a neurally modulated central regulator of systemic inflammation (Zaaqoq et al., 2014). Further computational studies on the systemic inflammatory responses of critically ill trauma patients have uncovered a novel, chemokine-based inflammation control circuit involving IP-10, along with the chemokines monokine induced by gamma interferon (MIG/CXCL9) and monocyte chemotactic protein-1 (MCP-1/CCL2) upstream of IL-6 (Azhar et al., 2021).

Here, we hypothesized that the tripartite network involving IP-10, MIG, and MCP-1, along with the cytokine IL-6, would be involved in cross-tissue inflammation regulated by the vagus nerve. To test this hypothesis, we leveraged a systems biology approach using hypergraphs–utilized previously to define novel roles of toll-like-receptor 4 (TLR4) in cross-compartmental inflammation following trauma and hemorrhagic shock, the role of the central nervous system in modulating wound healing, the role of the peripheral nervous system in modulating transplant rejection, and the role of the peripheral nervous system in modulating the gut microbiome (Mi et al., 2011; Shah et al., 2022; Shah et al., 2023)–to gain novel insights into the effect of vagotomy on cross-tissue inflammation.

## 2 Materials and methods

### 2.1 Experimental design

All experimental protocols were approved by the Institutional Animal Care and Use Committee at the Feinstein Institute for medical research. Adult C57BL6 mice were subjected to no surgery (baseline), sham surgery, or subdiaphragmatic vagotomy. In brief, mice were anaesthetized via intraperitoneal administration of a ketamine (100 mg/kg)/xylazine (10 mg/kg) cocktail. A midline incision was made, and the stomach was retracted inferiorly to expose the distal esophagus and the gastroesophageal junction. The anterior (left) and posterior (right) branches of the vagus nerve were identified running alongside the esophagus and severed distal to the hepatic branches. The stomach was then placed back into the anatomical position and the peritoneum was closed with a running 4–0 vicryl suture and surgical staples. For non-vagotomized mice (sham surgery group), the vagus nerve was gently exposed without further manipulation. Mice were allowed to recover for 7 days prior to additional experimentation.

Seven days after surgical intervention, mice were euthanized and heart, liver, lung, kidney, spleen, gut, and plasma samples were collected. Seven days post-surgery was selected as the endpoint of experimentation as it was considered to be a sufficient interval from the surgery to allow the acute post-surgical inflammatory response to subside.

Separately, mice that received sham surgery or subdiaphragmatic vagotomy were challenged with Gram-negative bacterial lipopolysaccharide (LPS), 7 days after surgery. Mice were treated with 3 mg/kg of LPS and were sacrificed 3 h after LPS challenge. Heart, liver, lung, kidney, spleen, gut, right and left brain, and plasma samples were collected. Overall, there were 10 mice in each of the four different experimental conditions.

### 2.2 Quantification of inflammatory mediators

Blood was collected via cardiac puncture and placed in tubes containing 10 units of sodium heparin, inverted 8–10 times, and then centrifuged to obtain plasma. All organs were rinsed with sterile PBS and then stored in 800uL of RNAlater overnight at 4°C and then transferred to −20 °C for storage until assayed. The following cytokines and chemokines were quantified using Luminex™ technology in all tissue and plasma samples: granulocyte/macrophage colony-stimulating factor (GM-CSF), interferon gamma (IFN-γ), interleukin (IL)-1α, IL-1β, IL-2, IL-4, IL-5, IL-6, IL-10, IL-12p40, IL-12p70, IL-13, IL-17A, inducible protein of 10 kDa (IP-10/CXCL10), keratinocyte-derived chemokine (KC/CXCL1), monocyte chemotactic protein-1 (MCP-1/CCL-2), Macrophage inflammatory protein-1 (MIP)-1α, monokine induced by gamma interferon (MIG/CXCL9), Vascular Endothelial Growth Factor (VEGF), and tumor necrosis factor-α (TNF). The expression of each inflammatory mediator was expressed in pg/mg of protein in tissue and pg/mL in plasma samples. To remove outliers, individual inflammatory mediator expression levels that were outside of 2 standard deviations from the mean inflammatory mediator expression levels in each tissue were omitted.

### 2.3 Statistical analysis: analysis of variance (ANOVA)

One-way ANOVA with *post hoc* multiple comparison analysis was conducted to quantify significant differences in inflammatory mediator expression levels among baseline, sham, and vagotomy conditions. Tukey’s honestly significant different procedure was used to compare cytokine expression levels across experimental conditions. This analysis was conducted for each inflammatory mediator in each tissue.

### 2.4 Dynamic network analysis (DyNA)

DyNA (Mi et al., 2011) was used to describe intra-tissue networks. Within a tissue, a *t*-test was conducted to assess for significant changes in inflammatory mediator expression levels following sham surgery or vagotomy compared to baseline (*p* < 0.05). A cross–correlational analysis of all significantly changing inflammatory mediators using Pearson’s correlation was conducted within each tissue. Inflammatory mediators that were significantly correlated with each other (*|r|* > 0.90) were deemed important intra-tissue connections.

### 2.5 Dynamic hypergraphs (DyHyp)

The dynamic hypergraph formalism was adapted to model the rate at which the expression of inflammatory mediators changed following sham surgery or vagotomy across tissues compared to baseline. In this model, tissues served as nodes and inflammatory mediators served as edges, as detailed previously (Shah et al., 2022). Unlike typical network inference approaches in which an edge connects only two nodes, DyHyps allow an edge to connect two or more nodes. This geometric flexibility of the DyHyp edge allows for the representation of dynamic inflammatory mediators across multiple tissues/compartments.

To determine if an inflammatory mediator qualified as an edge in the DyHyp model, a rate-of-change condition was applied to each inflammatory mediator in each tissue. Within a single tissue, the rate of change in expression of an inflammatory mediator was calculated between baseline and 7-day post sham surgery or vagotomy according to the following formula: $\upsilon_{A}=\frac{\left(Median\;{\left[A\right]}_{day\;7}-\;Median\;{\left[A\right]}_{day\;0}\right)}{7\;days}$ , where $\upsilon_{A}$ = the velocity (rate-of-change) of theoretical inflammatory mediator A. Following LPS challenge, the velocity of each inflammatory mediator was calculated according to: $\upsilon_{A}=\frac{\left(Median\;{\left[A\right]}_{3h\;post\;LPS\;}-\;Median\;{\left[A\right]}_{7d,0h\;after\;surgery}\right)}{3\;hours}$ .

Mediators that had a positive rate-of-change were separated from those that had a negative rate-of-change. Relative to the mean rate-of-change of mediators that were increasing with time, mediators whose rate-of-change was one standard deviation above the average rate of change were identified as positive edges encompassing the tissue (node) in which that inflammatory mediator was increasing. Likewise, mediators that were decreasing with time and were one standard deviation below that average rate-of-change of mediators that were decreasing were identified as negative edges encompassing the tissue.

Selection of mediators as edges encompassing a tissue using the rate-of-change threshold was conducted independently for each tissue. If two or more tissues had an edge in common, then an edge was drawn to encompass both nodes. DyNA networks were superimposed onto the DyHyp structure for easy visualization of both intra-tissue (DyNA) and cross-tissue (DyHyp) inflammatory networks. Inflammatory mediators that were correlated with each other were listed within the DyHyp node (tissue) and connected by an arrow. All computational DyHyp, DyNA, and rate-of-change models have been made publicly available at https://github.com/aShah-MDinTraining/DyHyp_RateOfChange.git.

### 2.6 Mechanistic computational modeling of the long-term effects of vagotomy on systemic inflammation

The Boolean network adapted for biological modeling (Albert et al., 2008) and response to trauma (Azhar et al., 2021) was modified to model the effects of vagotomy on systemic inflammation. The prior “chemokine switch” model, developed by Azhar et al., allowed for the feed-forward development of connections among MIG, MCP-1, IP-10, and IL-6 that simulated the evolution of systemic inflammation based on injury severity (Azhar et al., 2021). The “chemokine switch” model was based on a set of rules in which nodal connections were made based on patterns of cytokine and chemokine feedback inferred through dynamic Bayesian networks that modeled the evolution of inflammation in trauma patients. The model utilizes a node “x” whose behavior is stimulated by trauma and results in the upregulation of IP-10. It has been previously thought that node “x” has a pattern of behavior akin to that of IFN-γ in response to trauma (Azhar et al., 2021).

We tested the potential for the “chemokine switch” model to simulate the inflammatory response to sham surgery. We adapted this model to predict the inflammatory response in the absence of MIG, a mediator whose systemic response was diminished as a result of vagotomy, to model the effect of vagotomy. Rules influencing the nodal connections that affected the expression of MIG or that were dependent on the expression of MIG were removed. Variability is simulated in the Boolean model through random seeding of the initial network and repeated simulation, as detailed in Azhar et al.; specifically, the model was run 1,000 times and the average and variance in inflammatory mediator expression across all simulations is plotted (Azhar et al., 2021). The model predicted long-term inflammatory outcomes to vagotomy using a set of rules that did not facilitate intra-nodal connections between MCP-1, IP-10, and IL-6 with MIG.

The following rules, adapted from Azhar et al. (Azhar et al., 2021), were used to model the effect of vagotomy:

• IFNγ is elevated in the absence of severe injury or MCP-1.
• IP-10 is induced by IFNγ and has positive self-feedback, but the non-zero expression of MCP-1 can suppress IP-10.
• MCP-1 is induced by MCP-1 itself as long as IP-10 is not elevated.
• IL-6 is induced by MCP-1 and elevated IL-6 is suppressed by elevated IP-10.

## 3 Results

### 3.1 Differential expression of inflammatory mediators in multiple tissues following vagotomy

Our overarching hypothesis is that the vagus nerve is involved in the regulation of inflammation in multiple tissues, as well as in the maintenance of appropriately compartmentalized inflammation. As an initial test of this hypothesis, we determined the impact of sub-diaphragmatic vagotomy (vs sham surgery, control) on 20 protein-level inflammatory mediators in the heart, lung, liver, kidney, spleen, gut, and plasma of C57Bl/6 mice. The resultant dataset, after removing outliers for each inflammatory mediator in individual tissues, included tissue and plasma samples from the following experimental groups: baseline (no surgery) (*n* = 5), sham surgery (*n* = 26–30), and vagotomy (*n* = 25–30).

**FIGURE 1:**
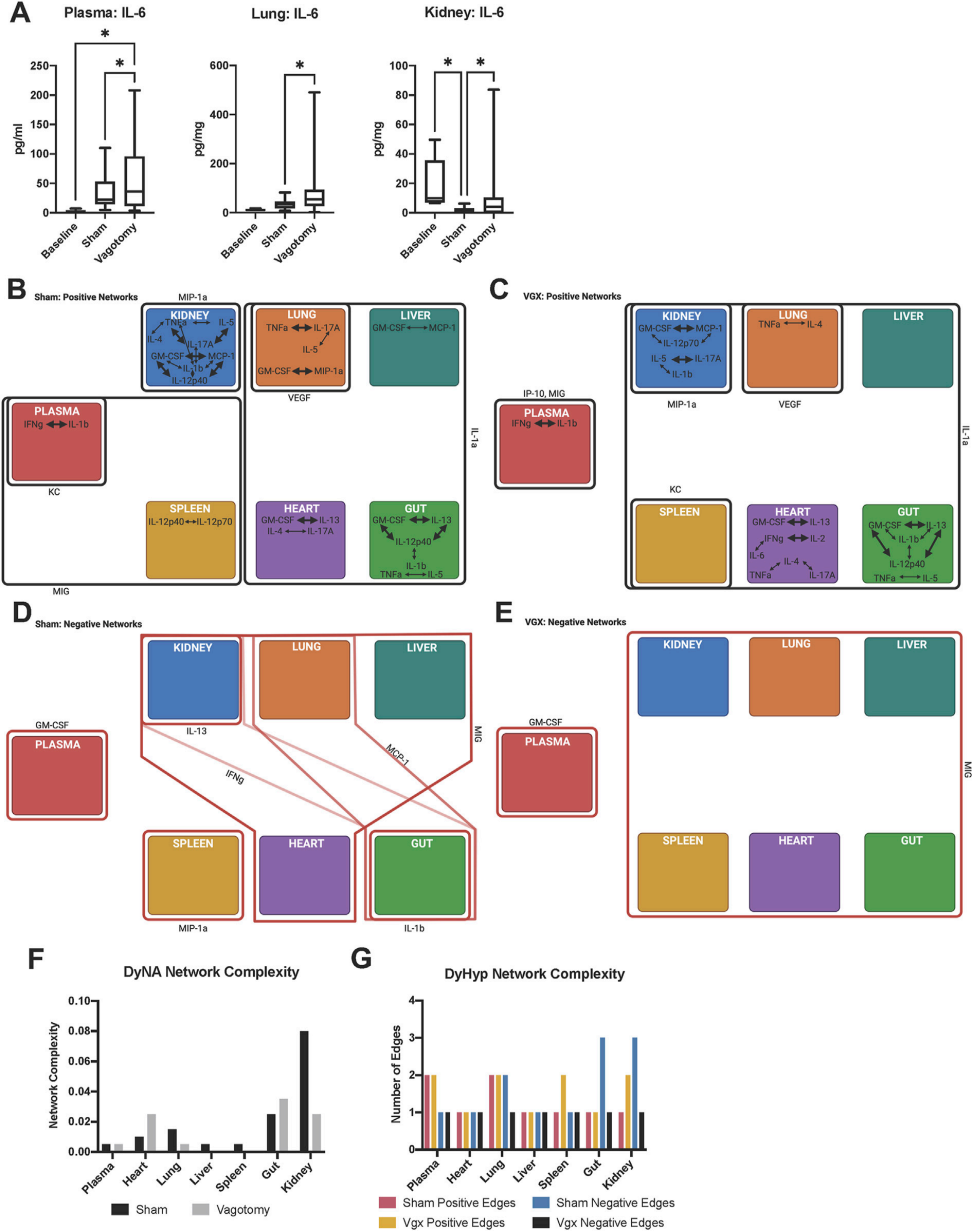
Inter-tissue inflammatory network analysis 7-day after sham surgery or vagotomy. **(A)** IL-6 is significantly elevated in the plasma, lung, and kidney following vagotomy compared to sham surgery. The edges of the box-and-whisker plot show data at the 25th and 75th percentile and the whiskers extend to the most extreme data points. **(B–E)** DyHyp nodes (tissue) are indicated by colored boxes. Edges surrounding one or more nodes are indicated as solid lines around nodes. Black lines indicate that the edge has a positive rate of change and red lines indicate that the edge has a negative rate-of-change. Intra-tissue networks (DyNA) are depicted within each DyHyp node to indicate that the inflammatory network is occurring within a single tissue. Thick black arrows indicate that inflammatory mediators are significantly positively correlated (*r* ≥ 0.95) and thin black arrows indicate that inflammatory mediators are positively, but not significantly, correlated (*r* > 0.90). **(F)** DyNA network complexity within each organ by experimental condition. **(G)** Graph of edge distribution, which indicates the number of edges surrounding each node in the DyHyp model.

The expression of most inflammatory mediators increased in several tissues following either sham surgery or vagotomy compared to baseline (Supplementary Figure S1). However, only four inflammatory mediators (MIG, IL-6, KC, and IL-1α) differed significantly in expression in more than one tissue between vagotomy and sham surgery (Supplementary Figure S1). The most robust changes included a decrease in MIG in every tissue assessed as well as the systemic circulation (plasma) following vagotomy compared to sham surgery. Furthermore, IL-6 was elevated in the plasma, kidney, and lung following vagotomy compared to sham surgery (Figure 1A, Supplementary Figure S1). Inflammatory mediators that induce the expression of MIG and IL-6, including IFNγ and CXCL10/IP-10, were elevated relative to baseline in most tissues following both sham surgery and vagotomy, suggesting that vagotomy differentially affected the extent of IFNγ− and IP-10-induced expression of MIG and IL-6 within each tissue.

### 3.2 Vagotomy is associated with the development of unique intra- and inter-tissue networks in all tissues

Given the effect of vagotomy on the expression of distinct inflammatory mediators in multiple tissues, we next hypothesized that the intra- and inter-tissue inflammatory networks would differ in mice following vagotomy compared to sham surgery. Furthermore, we hypothesized that the differences would largely pertain to MIG, IL-6, KC, and IL-1α, whose expression was significantly different between vagotomy and sham surgery. DyNA (Mi et al., 2011) was used to model intra-tissue inflammatory networks following vagotomy vs sham surgery, while DyHyps (Singh-Varma, Shah et al., *in review*) were used to define cross-tissue inflammatory networks. In DyNA, positive correlations among inflammatory mediators were interpreted as indicating the propagation of tissue-localized inflammation, whereas negative correlations implied the concerted downregulation of inflammation. DyHyp analysis was used to capture significant, dynamic features of inflammation across tissues. Specifically, inflammatory mediators whose rate of change relative to baseline was above or below 1 standard deviation of the average rate of change of that inflammatory mediator within a specific tissue were identified. DyHyp networks were constructed to highlight sets of tissues that, independently, exhibited the same salient dynamic changes as a means of inferring coordinated, cross-tissue inflammatory networks. The display of DyNA networks superimposed on DyHyp networks (Figures 1B–E) thus provides a simultaneous visualization of dynamic, intra- and inter-tissue propagation vs resolution of inflammation associated with vagotomy vs sham surgery.

Contrary to the hypothesis derived from our initial statistical analyses, intra-tissue inflammatory networks following both sham surgery and vagotomy did not involve MIG, KC, or IL-1α (Figures 1B, C). Interleukin-6 was correlated with IFN-γ following vagotomy in the heart, but aside from this single connection IL-6 was also absent from all other inter-tissue networks (Figure 1C). Instead, multiple tissues expressed networks involving IFN-γ, IL-1β, TNF-α, GM-CSF, and MIP-1α following surgical insult (Figures 1B, C).

This paradoxical finding raised the possibility that the statistically relevant impact of vagotomy on the expression of inflammatory mediators might arise from cross-tissue interactions. We therefore next utilized DyHyp to assess the role of the vagus nerve in cross-tissue propagation vs resolution of inflammation. We hypothesized that the inter-tissue expression of MIG, KC, and/or IL-1α might be affected by vagotomy. Moreover, we hypothesized that the vagus nerve was sensitive to changes in inflammation over time rather than the rapid induction and subsequent resolution of inflammation following surgical insult in each tissue. In support of this hypothesis, DyHyp analysis pointed to distinct intra-tissue trends in the expression of MIG, KC, and IL-1α between sham surgery and vagotomy. MIG expression increased concordantly in both the spleen and plasma following sham surgery (Figure 1B). Vagotomy appeared to sever the parallel, significant rise in MIG expression in both the spleen and plasma since, following vagotomy, MIG increased significantly in only the plasma but not the spleen (Figure 1C). Compared to sham surgery (Figure 1B), vagotomy was also associated with a simultaneous increase in IL-1α expression in all tissues (Figure 1C). Interestingly, KC and IP-10 exhibited elevated rates of change in the plasma, but not other tissues, following both sham surgery and vagotomy (Figures 1B, C).

DyHyp analysis suggested that vagotomy primarily disrupted the cross-tissue attenuation of inflammation following injury (compare Figures 1D, E to Figures 1B, C). Following sham surgery, but not vagotomy, coordinated downregulation of IL-13, IFN-γ, MCP-1, MIP-1α, and IL-1β was inferred in one or more tissue compartments. The downregulation of IFN-γ in both the kidney and gut as well as that of MCP-1 in both the lung and gut suggested the existence of vagally connected kidney-gut and lung-gut axes (Figure 1D). Following vagotomy, MIG was downregulated in all tissues, including the spleen and gut, in contrast to sham surgery (Figures 1D, E).

### 3.3 Differences in intra- and inter-tissue network complexity following vagotomy

Following vagotomy, intra-tissue DyNA network complexity was reduced in the kidney and lung but elevated in the heart and gut relative to sham surgery (Figure 1F). Cross-tissue (DyHyp) network complexity analysis suggested that pro-resolution networks, as indicated by negative DyHyp edges, were less complex following vagotomy than those discerned following sham surgery (Figure 1G). Furthermore, this analysis suggested a decrease in the pro-resolution DyHyp network complexity in the gut, kidney, and lung following vagotomy compared to sham surgery (Figure 1G), suggesting that the vagus nerve may be a key driver of the downregulation of inflammation across the gut-kidney and gut-lung axes. The spleen and liver contained no significant intra-tissue inflammatory networks following vagotomy unlike sham surgery, in which intra-tissue networks were developed in both organs.

### 3.4 Rate-of-change modeling suggests a novel, inter-tissue, vagally regulated inflammatory threshold involving the spleen and impacting IFNγ, IP-10, MIG, MCP-1, and IL-6

ANOVA, DyNA, and DyHyp analyses suggested that vagotomy differentially affects either the steady-state levels and/or rate of change of IFNγ, IP-10, MIG, MCP-1, and IL-6 across one or more tissues. While IFN-γ, MIG and MCP-1 were inferred as components of inter- and/or intra-tissue networks across multiple tissues, IP-10 was a unique edge in the systemic circulation network following vagotomy but not sham surgery. Interleukin-6 was not a significant inter- or intra-inflammatory edge in multiple tissues but was elevated significantly following vagotomy relative to sham surgery in the plasma, kidney, and lung.

**FIGURE 2:**
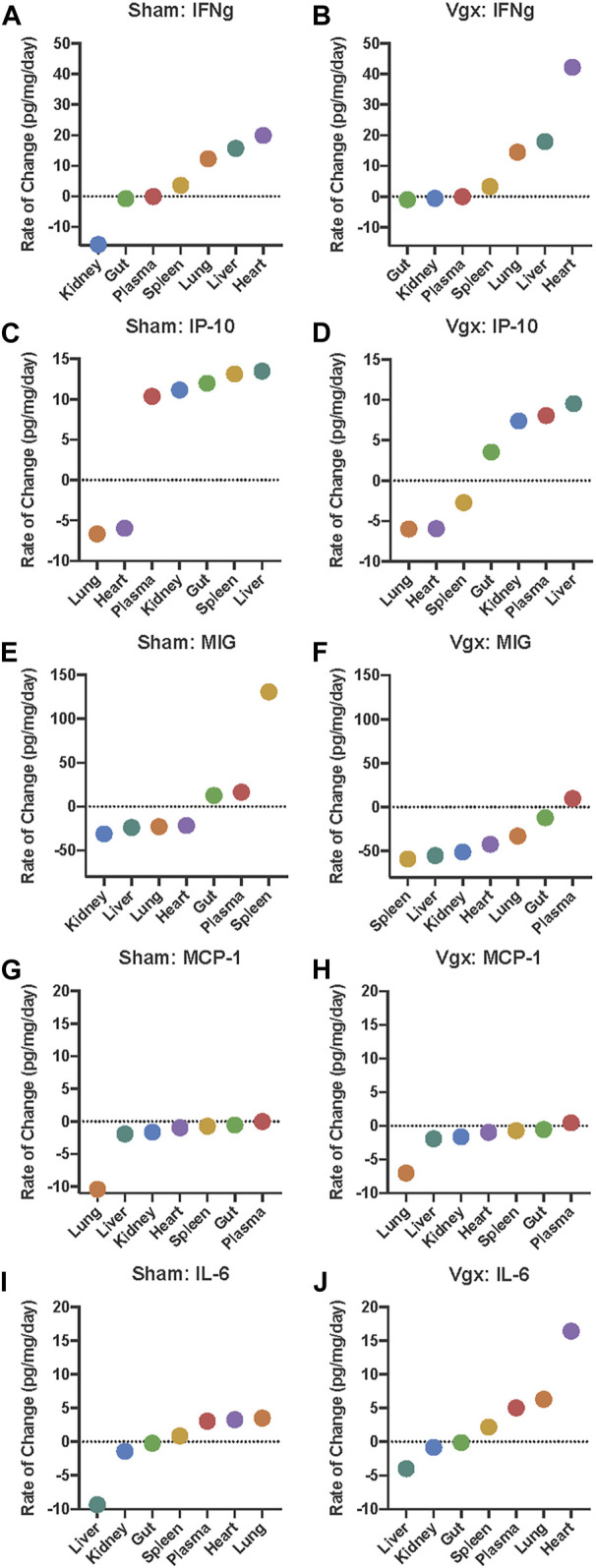
Rate of change analysis of IFN-γ, IP-10, MIG, MCP-1, and IL-6 in distinct organs and the systemic circulation. **(A–J)** The rate of change for each inflammatory mediator was calculated in each organ. Organs are organized on the *x*-axis by increasing rate-of-change for the respective inflammatory mediator graphed. Negative rates-of-change indicate that the expression of the inflammatory mediator is decreasing with time whereas positive rates-of-change indicate that the expression of the inflammatory mediator is increasing with time.

As a potential means of explaining these disparate findings, we hypothesized that the dynamic spatiotemporal expression of IFN-γ and subsequent expression of the IFN-γ-induced chemokines IP-10, MIG, and MCP-1 may influence the rate of change of IL-6 and account for its unique spatiotemporally sensitive expression. We therefore sought to assess for the rate of change rather than absolute change as the absolute change could vary more significantly by tissue. We plotted the rate of change of these mediators in rank order across all assessed tissues, hypothesizing that this analysis might identify features consistent with tissue-specific, regulatory thresholds. The rate of change in inflammatory mediator concentration in each tissue was plotted from least to greatest rate. In doing so, the graphs assumed a sigmoidal shape, consistent with the existence of a biological threshold, which upon being crossed, results in the acceleration of mediator expression across subsequent tissues. Notably, the ordering of these tissues along the *X*-axis for each cytokine varied by both inflammatory mediator studied and experimental condition. In this analysis, a sigmoidal sequence of rates of change in various tissues spanning across a rate of zero (i.e., no change relative to baseline expression) served as a means of defining the tissue(s) involved in such a putative threshold (Figure 2).

Analysis of the rate of change of IFN-γ across all tissues pointed to differential expression of this cytokine primarily in the kidney and heart in vagotomy vs sham surgery. Following sham surgery, IFN-γ decreased in expression at a rate of 15.7 pg/mg/day in the kidney whereas, following vagotomy, IFNγ decreased more slowly (0.6 pg/mg/day) (Figures 2A, B). Aside from the kidney and gut, IFN-γ increased nearly twice as fast in the heart following vagotomy compared to sham surgery (Figures 2A, B). In all other tissues, the rate of change of IFN-γ following sham surgery and vagotomy was similar.

Interferon-γ is the key cytokine that induces IP-10 (“IFN-γ-inducible protein of 10 kDa”) (Luster et al., 1985). Rate-of-change models highlighted that the magnitude and direction of change in IP-10 differed only in the spleen between vagotomy and sham surgery (Figures 2C, D). This difference was striking: following sham surgery, IP-10 increased in the spleen at a rate of 13.1 pg/mg/day (Figure 2C); in contrast, IP-10 decreased at a rate of −2.7 pg/mg/day following vagotomy (Figure 2D). In support of our threshold hypothesis, the sequence of rates of change in various tissues of IP-10 followed a sigmoidal curve with an inflection point that appeared roughly at the point y = 0 following both sham surgery and vagotomy, with vagotomy appearing to blunt this sigmoidal transition compared to sham surgery (Figures 2C, D). This pattern was absent when compared to the rate-of-change graphs of IFN-γ, MIG, MCP-1 and IL-6.

The unique location of the of the inflection point at y = 0 suggests the existence of a biological threshold whereby tissues located above the threshold increase IP-10 expression and tissues located below the decrease IP-10 expression. Following sham surgery, the rate of change of IP-10 in the spleen occurred above the inflection point; however, following vagotomy, the rate of change of IP-10 in the spleen was inferred as occurring below the inflection point (Figures 2C, D). This pattern was not observed for IP-10 in any other tissue examined.

Since IP-10 and MIG share CXCR3 as a receptor (Richardson et al., 1995; Richardson et al., 1998; Richardson et al., 2000; Nasser et al., 2005; Paust et al., 2012; Giego et al., 2013), we examined the spatiotemporal dynamics of MIG next. Like IP-10, MIG decreased in the spleen following vagotomy (−59.0 pg/mg/day) in contrast to a marked increase (130.7 pg/mg/day) following sham surgery (Figures 2E, F). A similar pattern was also observed in the gut, in general support of the hypothesis that the spleen may serve as a vagally sensitive, rate-determining gauge for the dynamic changes in MIG expression in the other tissues sampled. Finally, while MIG expression declined in the liver, kidney, heart, and lung, the rate of decline was faster following vagotomy compared to sham surgery.

We have implicated IP-10, MIG and MCP-1 in a cross-regulatory network that ultimately impacts systemic IL-6 following traumatic injury in humans (Azhar et al., 2021). Accordingly, we next examined the tissue-specific rate of change of MCP-1 and IL-6. This analysis suggested that tissue-specific changes in MCP-1 are similar following both sham surgery and vagotomy (Figures 2G, H), suggesting that this central pro-inflammatory chemokine is not regulated via the vagus nerve. In contrast, the shape and tissue-specific expression sequence of the rate-of-change graphs of IL-6 differ following vagotomy vs sham surgery, with a deviation associated with the rapid rise of IL-6 in the heart following vagotomy (Figures 2H–J).

### 3.5 Vagotomy alters IL-6- and MCP-1-mediated intra-tissue inflammatory networks in response to LPS challenge

We next examined the potential impact of the inferred, vagus-mediated regulation of cross-tissue inflammation following injury by subjecting mice to a subsequent pro-inflammatory stimulus, Gram-negative bacterial lipopolysaccharide (LPS), a potent microbial mediator in the pathogenesis of systemic inflammation in sepsis and septic shock (Opal, 2010; Tan and Kagan, 2014) that also serves as a means of inducing a quantifiable systemic acute inflammation (Parker and Watkins, 2001). Following LPS challenge, multiple splenic and hepatic inflammatory mediators were elevated in the context of either vagotomy or sham surgery, including IFNγ, KC, IL-6, MCP-1, MIG, and IP-10 in the spleen and GM-CSF, IFN-γ, IL-1α, IL-2, IL-10, IL-12p70, IL-13, and IL-17A in the liver (Supplementary Figures S2, S3). Furthermore, cardiac IFN-γ expression decreased significantly in vagotomized mice following LPS challenge but not in mice that had received sham surgery (Supplementary Figures S2, S3).

**FIGURE 3:**
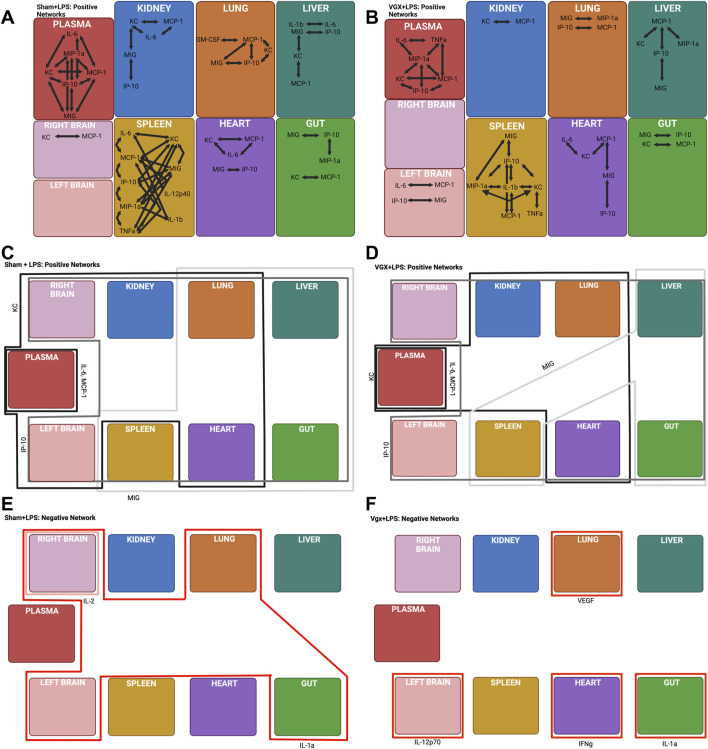
DyNA and DyHyp models of inter- and intra-tissue inflammation in sham and vagotomized mice in response to LPS challenge. **(A, B)** DyNA networks highlight intra-tissue correlations between inflammatory mediators (*|r|* > 0.95) in mice that had received sham surgery and were subsequently challenged with LPS. **(C–F)** DyHyp inflammatory networks highlight inter-tissue trends in inflammatory mediators whose rate-of-change is outstanding across one or more tissues.

DyNA highlighted prominent differences between the intra-tissue networks in the spleen and plasma, but not the liver (Figures 3A, B) when LPS was administered following vagotomy vs sham surgery. Tumor necrosis factor-α is, a key, LPS-inducible, pro-inflammatory cytokine whose expression is canonically negatively regulated by the vagus nerve (Tracey, 2002). This cytokine was observed only in inflammatory networks of vagotomized mice subjected to LPS, remaining connected with IL-6, MIP-1α, and MCP-1 (Figure 3B). Within the spleen, intra-tissue network complexity was greater in the spleen in the sham + LPS group compared to the vagotomy + LPS group (Supplementary Figure S4A). The difference in network complexity appeared to be related to the presence of IL-6, which was often correlated with MCP-1, in the inflammatory networks of mice that underwent sham surgery + LPS (Figure 3A). Interleukin-6 was absent from the intra-spleen inflammatory networks in the vagotomy + LPS condition even though IL-6 was elevated significantly following LPS challenge in both vagotomy sham surgery groups (Figure 3B, Supplementary Figures S2, S3).

We have demonstrated previously that MCP-1 stimulates IL-6 expression in stressed hepatocytes, with potential impact on systemic inflammation in human blunt trauma patients (Ziraldo et al., 2013). Our analyses suggested that the control of IL-6 by MCP-1, specifically in the heart, was disrupted following vagotomy alone (Figures 2G–J). In contrast, based on DyNA, IL-6 was not correlated with MCP-1 expression in any tissue in vagotomized mice challenged with LPS, suggesting a disrupted co-regulation of IL-6 by MCP-1 (Figure 3B).

### 3.6 Vagotomy limits the increase in MIG expression in response to LPS challenge in the lung and heart

We next examined the impact of LPS on intra- and inter-tissue dynamic networks. Following challenge with LPS, MIG was absent from the intra-tissue inflammatory network in the plasma of vagotomized mice but not those in mice that received sham surgery (Figures 3A, B). MIG was connected with IP-10, MIP-1α, MCP-1, and KC in the systemic circulation of mice that had received sham surgery (Figure 3A), suggesting that the vagally regulated expression of MIG had effects on the tissue-specific expression of other cytokines and chemokines following LPS challenge.

Inter-tissue analysis (DyHyp network complexity) highlighted differences in complexity in the heart, lung, and brain between experimental conditions (Supplementary Figure S4B). This analysis suggested that the vagus nerve is involved in facilitating the propagation of LPS-induced intra-tissue inflammation between multiple organs and the heart, lung, and brain. Distinct organs exhibited increased MIG following LPS challenge in mice undergoing vagotomy vs those undergoing sham surgery (Figures 3C, D). The prior DyHyp and rate-of-change graphs modeling inflammation following vagotomy alone (Figure 2F) suggested that vagotomy results in the downregulation of MIG in the spleen and this downregulation affects the rate of increase of MIG in other tissues. MIG was upregulated in the spleen, liver, gut, heart, and lung following LPS alone. In contrast, MIG was only upregulated in the spleen, liver, and gut in the vagotomy + LPS condition. We inferred that, following LPS challenge, the pro-inflammatory response initiated by the spleen was insufficient to upregulate MIG substantially in the heart and lung in the absence of the vagus nerve (Figure 3D). In contrast to the spatiotemporal dynamics of MIG expression, LPS-induced IP-10 upregulation was observed across all tissues, independent of the rate of IP-10 upregulation in the spleen, in both vagotomized mice and mice that underwent sham surgery (Figures 3C, D).

Vagotomy also appeared to inhibit other facets of the inter-tissue downregulation of inflammation. Interleukin-1α was downregulated in the gut alone following vagotomy and LPS but was downregulated in the gut, left brain, right brain, and lung following sham surgery + LPS (Figures 3E, F). There was no inter-tissue connectivity for IL-1α in vagotomized mice, while this cytokine was the sole cross-tissue inflammatory mediator in the sham + LPS condition (Figures 3E, F). Furthermore, while no cross-tissue inflammatory mediators were downregulated in the vagotomy + LPS condition, IFN-γ was downregulated significantly in the heart following vagotomy + LPS relative to sham surgery + LPS (Figures 3E, F).

### 3.7 Lipopolysaccharide differentially affects the rate of expression of inflammatory mediators based on the mediator itself, the tissue affected, and vagal innervation

**FIGURE 4:**
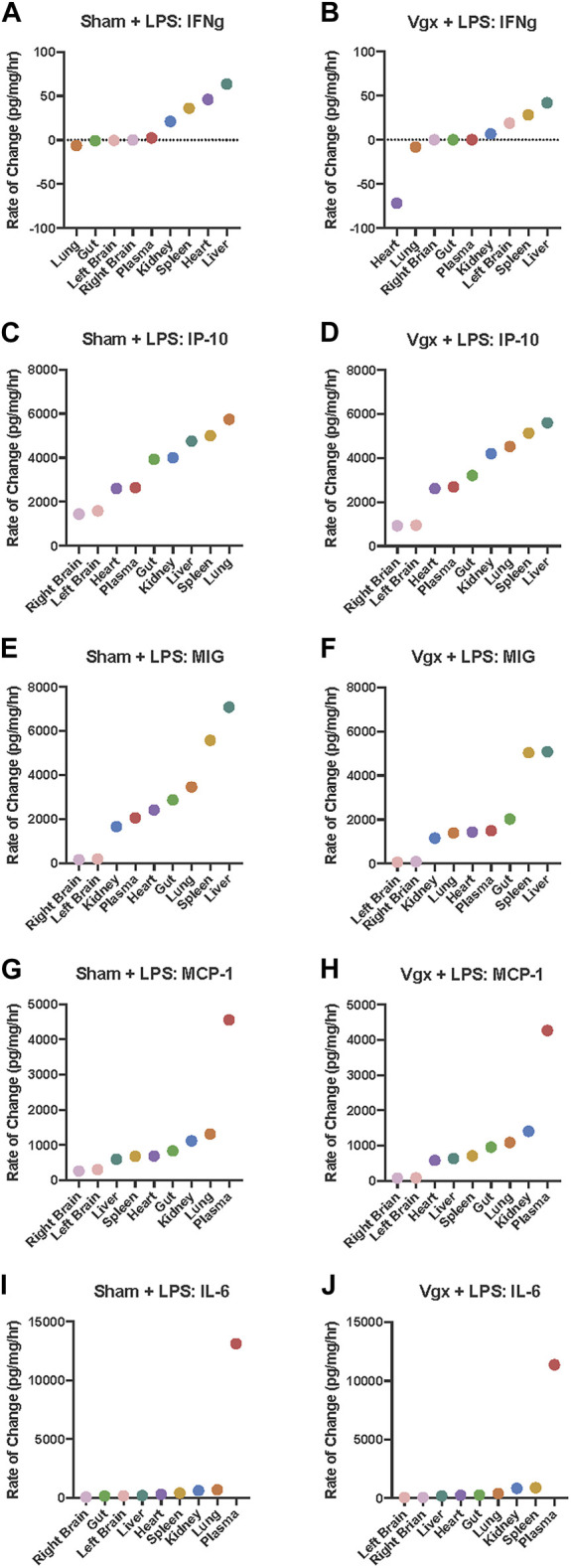
Rate-of-change of IFN-γ, IP-10, MIG, MCP-1 and IL-6 in tissues following LPS challenge in mice previously subjected to sham surgery or vagotomy. **(A–J)** The rate of change of inflammatory mediator expression in response to LPS challenge was calculated. Organs are organized on the *x*-axis by increasing rate-of-change for the respective inflammatory mediator graphed.

In parallel with our prior analyses of vagotomy or sham surgery alone (Figure 2), we next utilized a rate-of-change modeling to examine the impact of LPS subsequent to vagotomy vs sham surgery. Following sham surgery + LPS challenge, the slope of the rise of IFNγ between the kidney, spleen, heart, and liver, was more steep than the slope of the rise of IFNγ between the kidney, spleen, and liver following vagotomy + LPS (Figures 4A, B). Furthermore, IFNγ decreased, rather than increased, in the heart of previously vagotomized mice (Figure 4B).

In contrast to surgical injury alone, a setting in which the vagus was inferred to regulate both IP-10 and MIG, the addition of LPS induced the rapid upregulation of IP-10 (Figures 4C, D) and MIG (Figures 4E, F) across multiple tissues in a vagus-independent manner. However, following vagotomy + LPS but not sham surgery + LPS, there was a large disjoint between the rate of change of MIG expression in the spleen vs that in the plasma, gut, heart, and lung (Figures 4E, F). The slope of the rate of change of MIG between the kidney, plasma, heart, gut, and lung following sham surgery + LPS was much steeper than the rate of change in MIG in the same set of organs following vagotomy + LPS. Overall, the rate-of-change model indicated that MIG increased more rapidly throughout organs in the sham surgery + LPS condition compared to the vagotomy + LPS condition. While the rate of change of MIG in the spleen appeared to dictate the rate of MIG regulation in other tissues, as inferred previously (Figures 2E, F), following vagotomy and LPS challenge, the rate-of-change of MIG in the spleen did not sufficiently upregulate the rate of MIG expression in the heart and lung (Figure 3D).

Analysis of intra-tissue inflammatory networks highlighted that, in response to vagotomy and LPS, pro-inflammatory networks in the plasma, heart, spleen, and kidney did not involve the correlated expression of IL-6 and MCP-1 that was inferred in those tissues following sham surgery + LPS (Figures 4G–J). Rate-of-change graphs showed that the exponential shape of the graph of inter-tissue propagation of IL-6 was the same as the exponential shape of the graph of MCP-1, following both vagotomy and LPS and sham surgery and LPS. Furthermore, the rate-of-change of IL-6 and MCP-1 in each tissue was similar between experimental conditions, suggesting that differences in the intra-tissue networks were not associated with the rate-of-change of MCP-1 and IL-6 in each tissue, but instead were due to the tissue-specific impact of vagotomy.

### 3.8 A boolean mechanistic model testing core hypothesis of vagus-regulated interaction among IFN-γ, IP-10, MIG, and IL-6 predicts that vagal regulation of MIG affects long-term expression of IP-10

The rich, complex, cross-tissue inflammatory dynamics and inferred threshold behavior described above suggest that the compendium of tissue compartments act as a single “system of systems.” As an initial test of the potential combined impact of the mechanisms inferred from data-driven computational analyses as well as predicting the longer-term impact of the inferred regulatory circuits involving IFN-γ, IP-10, MIG, MCP-1, and IL-6, we utilized a modified, previously described dynamic Boolean network model (Azhar et al., 2021), derived from dynamic network analyses of systemic inflammation in human blunt trauma patients. We hypothesized that this model could capture core effect of inter-tissue spread of inflammation on the systemic circulation despite the fact the model consists of a single, abstracted compartment representing the whole organism in response to blunt trauma (Azhar et al., 2021).

In the original “chemokine switch” model, IL-6 expression is induced by MIG or MCP-1 expression. Additionally, IP-10 expression is suppressed by MIG or MCP-1 expression but induced by its own upregulation (Supplementary Figure S5A). We hypothesized that the unmodified “chemokine switch” could capture the impact of sham surgery on systemic inflammation. We further hypothesized that an *in silico* MIG knock-out model could represent the impact of vagotomy. Accordingly, model rules were modified such that IL-6 expression occurred independently of MIG, and IP-10 expression was only suppressed by MCP-1 but not MIG (Supplementary Figure S5A).

**FIGURE 5:**
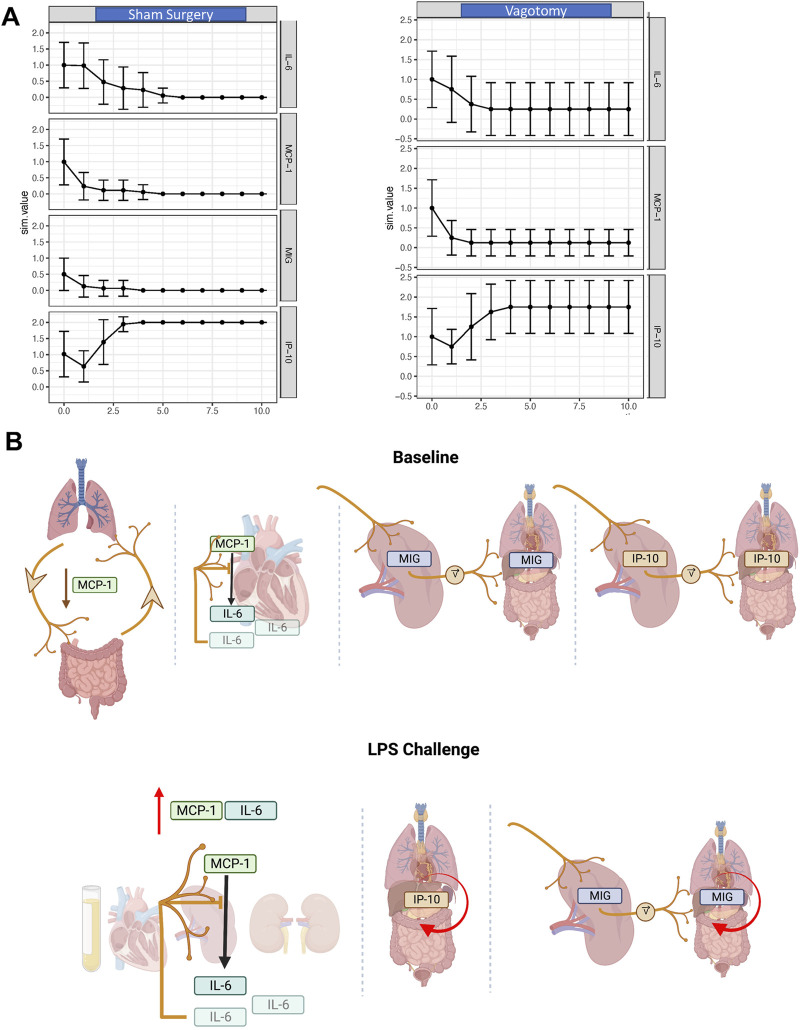
In silico and conceptual models of the long-term effects of vagotomy on IL-6, MCP-1, and IP-10 expression. **(A)** Inflammatory response to sham surgery and vagotomy as predicted by a Boolean network. The effect of vagotomy was modeled by excluding intra-nodal connections involving MIG. **(B)** A conceptual model of the inter-tissue impact of the vagus nerve inferred from experimental and computational analyses. At baseline, the vagus nerve downregulates MCP-1 across the lung-gut axis. In parallel, the rate of MIG and IP-10 expression across tissues is set by the vagally tuned rate of MIG and IP-10 expression in the spleen. Above a certain pro-inflammatory threshold induced by LPS, MCP-1 stimulates the expression of IL-6 and this is inhibited in the heart by the vagus nerve. Vagal innervation has minimal effect on the kinetics of IP-10 expression in response to LPS. Further, MIG expression across tissues is in part influenced by LPS as well as the vagally tuned rate of MIG expression by the spleen. In parallel, MCP-1 induces expression of IL-6 in the heart, spleen, and kidney and this cytokine also appears (or is expressed by cells in) the systemic circulation.

The “chemokine switch” and “chemokine switch with MIG KO” were used to simulate the temporal progression over 10 unitless time steps following sham surgery or vagotomy, respectively. While the abstract, compartment-less Boolean network did not capture the dynamics of IL-6 and MCP-1 (Figures 1, 3), the model did recapitulate the IP-10 response following both sham surgery and vagotomy. In concordance with Figure 2, model-simulated IP-10 exhibits a sigmoidal cross-tissue trajectory following both sham surgery and vagotomy (Figure 5A). Furthermore, IP-10 rises to a greater extent in the “sham” vs the “vagotomy” Boolean network (Figure 5A), just as it does in the rate-of-change model (compare Figures 2C, D). While vagotomy cannot be oversimplified to knocking-out MIG as doing so did not recapitulate the extent of systemic inflammatory changes observed *in vivo*, we can infer that vagotomy has a significant effect on MIG expression and the consequence of altered MIG expression affects subsequent IP-10 expression as evidenced in Figure 5A.

As in Figure 2, we hypothesize that the sigmoidal shape of the IP-10 trajectory suggests a threshold behavior, one that is attenuated following vagotomy. In further support of this hypothesis, Boolean network simulations also predict that, following vagotomy, IP-10 has greater variability in expression (larger error-bars) compared to sham surgery. The oscillatory variance of IP-10 following vagotomy suggests that the vagus nerve not only regulates IP-10 expression through upstream regulation of MIG, but that the vagus nerve also works to dampen the variance in IP-10 expression over time (Figure 5A). Finally, the Boolean network resulted in a transition state diagram with a lower degree of network complexity in the model of vagotomy compared to sham surgery (Supplementary Figure S5B). These relative differences in network complexity further suggest that the vagus nerve serves a key role in potentiating and regulating cross-tissue inflammation.

## 4 Discussion

In the present study, novel computational methodology was leveraged to gain new insights into the role of the vagus nerve in regulating inflammation both within and between tissues. Inflammatory mediators were quantified across tissues over time in mice that were subjected to subdiaphragmatic vagotomy or control sham surgery, and these data were used as input into computational models. Dynamic hypergraphs, which model the inter-tissue spread of inflammation were utilized in concert with dynamic network analysis, which models intra-tissue inflammatory networks, to create a Boolean “digital twin” model of that abstracts the inferred effects of the afferent arm of the inflammatory reflex on trans-compartmental inflammation. Furthermore, key hypotheses encoded–namely, that the vagus nerve was critical in the cross-compartmentalization of inflammatory mediators, that the cross-tissue spread of inflammation would be stifled following vagotomy, and that the spleen plays a unique role in modulating cross-tissue inflammation–were tested in the context of additional stimulation with LPS.

Taken together, these studies suggest a complex series of vagus-dependent and -independent processes (Figure 5B). At baseline, the vagus nerve appears to facilitate the downregulation of MCP-1 across the lung-gut axis. Following exposure to a potent pro-inflammatory stimulus, MCP-1 induced expression of IL-6 is inhibited in the heart by the vagus nerve. The rate of MIG and IP-10 expression across tissues is set by the vagally tuned rate of MIG and IP-10 expression in the spleen. Following LPS challenge, the MCP-1-induced expression of IL-6, above a threshold, in the plasma, heart, spleen, and kidney. Vagal innervation has minimal effect on the kinetics of IP-10 expression in response to LPS. MIG expression across tissues is in part influenced by LPS as well as the vagally tuned rate of MIG expression by the spleen.

The first set of experiments conducted modeled the impact of sham surgery and subdiaphragmatic vagotomy on the intra- and inter-tissue inflammatory responses. MIG, IL-6, KC, and IL-α were significantly different after vagotomy in multiple tissues. The rate-of-change of MIG and IP-10 were particularly sensitive to vagotomy in the spleen. Following vagotomy, MIG and IP-10 decreased in the spleen and the direction and magnitude of this decrease in the spleen appeared to affect the rate-of-change of MIG and IP-10 expression in the other tissues sampled. Conversely, MIG and IP-10 increased in the spleen following sham surgery, and this increase also uniquely affected the rate-of-change of MIG and IP-10 in the other tissues sampled. It appeared that both MIG and IP-10 served as vagally sensitive gauges in the spleen whose tuning affected the rate-of-change of MIG and IP-10 expression in other tissues following surgical injury. This unprecedented finding supports allusions to the role of IP-10 in regulating CD4^+^ T-cell expression in the spleen. These data support a nascent hypothesis that as the concentration of IP-10 in certain organs/tissues decreases (Figures 2C, D) and the rate of decrease becomes smaller and smaller in subsequent organs, there appears to be a “switch” in some cross-tissue communications at which point the rate of IP-10 increases rapidly in other compartments, including the plasma.

Prior work shows that mice subjected to cyclophosphamide-induced cystitis alongside an IP-10 inhibitor exhibited a decreased number of CD4^+^ T-cells compared to mice that did not receive an IP-10 inhibitor (Sakthivel et al., 2008). Thus, it is possible that changes in MIG and IP-10 expression in the spleen affect the rate of MIG and IP-10 upregulation in subsequent tissues via both the vagus nerve and splenic T-cells.

The mechanisms that mediate the changes in expression of these mediators between the spleen to other tissues following vagotomy are likely complex, especially given the differences observed between the spleen and systemic circulation. The spleen and liver are connected by the portal vein. The close connection between the two organs via the portal vein enables delivery of splenic cytokines and living cells to the liver (Elchaninov et al., 2023). Accordingly, changes in the expression of splenic cytokines may subsequently affect immune responses in the liver. An alternative possibility involves macrophages in other organs. Although the spleen is a major source of TNF and IL-1β production after LPS challenge (Huston et al., 2006), macrophages in organs such as the lung and/or liver may also respond to LPS, thus resulting in differential cytokine responses. Further studies are needed to address this important question.

The vagally responsive expression of inflammatory mediators such as IFN-γ, MCP-1, and IL-6 exhibited tissue-based effects. Interestingly, the rate-of-change of MCP-1 expression appeared to be independent of the vagus nerve, but the effect that MCP-1 had on controlling IL-6 expression in the heart appeared to be related to vagal innervation. Prior research suggests that MCP-1 induces the expression of IL-6 (Ziraldo et al., 2013). This body of work finds that in addition to MCP-1 inducing the expression of IL-6, the expression of IL-6 above a certain threshold results in the feedback-inhibition of MCP-1 induced expression of IL-6. Furthermore, dynamic network analysis suggests that this feedback-inhibition is dependent on the vagus nerve.

Dynamic network analysis also suggests that vagotomy perturbs the network consisting of the downregulation of MCP-1 across the gut-lung axis and the control of MCP-1 on IL-6 expression in the heart. This first set of studies appeared to highlight two related, but also different, inter- and intra-tissue inflammatory networks. One network consisted of the vagally sensitive expression of chemokines, IP-10 and MIG, in the spleen whose rate of expression set the pace for the rate of expression of IP-10 and MIG in other tissues. The second network consisted of MCP-1 and IL-6, whose inter- and intra-tissue expression was affected by vagotomy on a tissue-by-tissue basis. To understand how these two networks were altered in response to a potent inflammatory stimulus, mice that had previously undergone sham surgery or vagotomy were challenged with LPS and cross-tissue inflammatory networks were modeled once more. These models highlighted that the vagally sensitive and inter-tissue rate-determining expression of IP-10 in the spleen was abolished in response to a potent stimulus like LPS. In response to LPS, IP-10 expression was upregulated similarly across all tissues regardless of experimental condition. That is, the vagal regulation of IP-10 through the spleen could be trumped by a strong pro-inflammatory stimulus that would induce IP-10 expression across all tissues. Since the vagus nerve does not innervate the spleen directly, and that the afferent arm of the vagus nerve is through the sympathetic nervous system (not altered by subdiaphragmatic vagotomy), there may be a complex inflammatory mediator specific response via the spleen that occurs in response to LPS challenge (Pongratz and Straub, 2014).

In contrast, the vagally sensitive and inter-tissue rate-determining expression of MIG in the spleen was not totally abolished in response to LPS. MIG expression was upregulated in the spleen independent of the vagus nerve in response to LPS challenge. However, the ability of MIG to induce outstanding upregulation of MIG in the heart and lung was reduced in mice that had previously been vagotomized. This suggested, that even though LPS set the rate of MIG expression in the spleen, an intact vagus nerve was necessary to perpetuate the increase in MIG in organs such as the heart and lung.

The inter-tissue networks of MCP-1 and IL-6 suggested that vagotomy had minimal effect on the rate of change of these two inflammatory mediators in each tissue in response to LPS challenge. However, vagotomy prevented the positive intra-tissue correlation of MCP-1 and IL-6 in the plasma, heart, spleen, and kidney in response to LPS challenge. The hallmarks of this systems biology-derived model of the effects of vagotomy on regulating inter- and intra-tissue inflammatory networks are summarized in Figure 5A and Supplementary Table S1. We note that these inferences were limited by our experimental design, though supported by our prior work showing that MCP-1 regulates IL-6 (Ziraldo et al., 2013) and the finding that IL-6 was inhibited in various tissues in the vagotomy/LPS arm but not the sham/LPS arm. It is certainly possible that alternative explanations exist for our findings; in fact, given the myriad biological pathways that were not assessed in the present study, this is nearly a certainty.

This body of work has additional limitations as well. Future work should assess a larger number of data points and a wider set of inflammatory mediators and define the difference in intra- and inter-tissue inflammatory responses following subdiaphragmatic vagotomy, splenic nerve transection, and splenectomy. Also, the hypothesis that the inferred impact on T cells is mediated via the release of Ach must be tested explicitly. Finally, the mechanistic computational model should be expanded to include explicitly the tissue-specific manifestations of the “chemokine switch” involving IP-10, MIG, and MCP-1.

This intra- and inter-tissue model of the role of the vagus nerve in perpetuating and attenuating inflammation suggests nuanced vagally tuned roles for MIG and IP-10 expression in the spleen that affect MIG and IP-10 expression across tissues and throughout the systemic circulation. Furthermore, these studies suggest that the vagus nerve exerts tissue-specific effects on intra-tissue inflammatory networks, especially those mediated by IL-6. As the field of systems biology approaches a more complete development of robust “digital twin” models of the human body and its response to inflammation (Vodovotz, 2023), interdisciplinary studies using state-of-the-art omics data integrated via dynamic spatiotemporal models such as DyHyp and DyNA may help better delineate how inflammation is regulated in and across tissues via neural mechanisms.

## Data Availability

The original contributions presented in the study are included in the article/Supplementary Material; further inquiries can be directed to the corresponding author.
