# Supplementary figures and images for: Computational inference of chemokine-mediated roles for the vagus nerve in modulating intra- and inter-tissue inflammation
Source: Front Syst Biol. 2024 Feb 15;4:1266279. doi: 10.3389/fsysb.2024.1266279 (PMC12341964; doi:10.3389/fsysb.2024.1266279)

A

Plasma

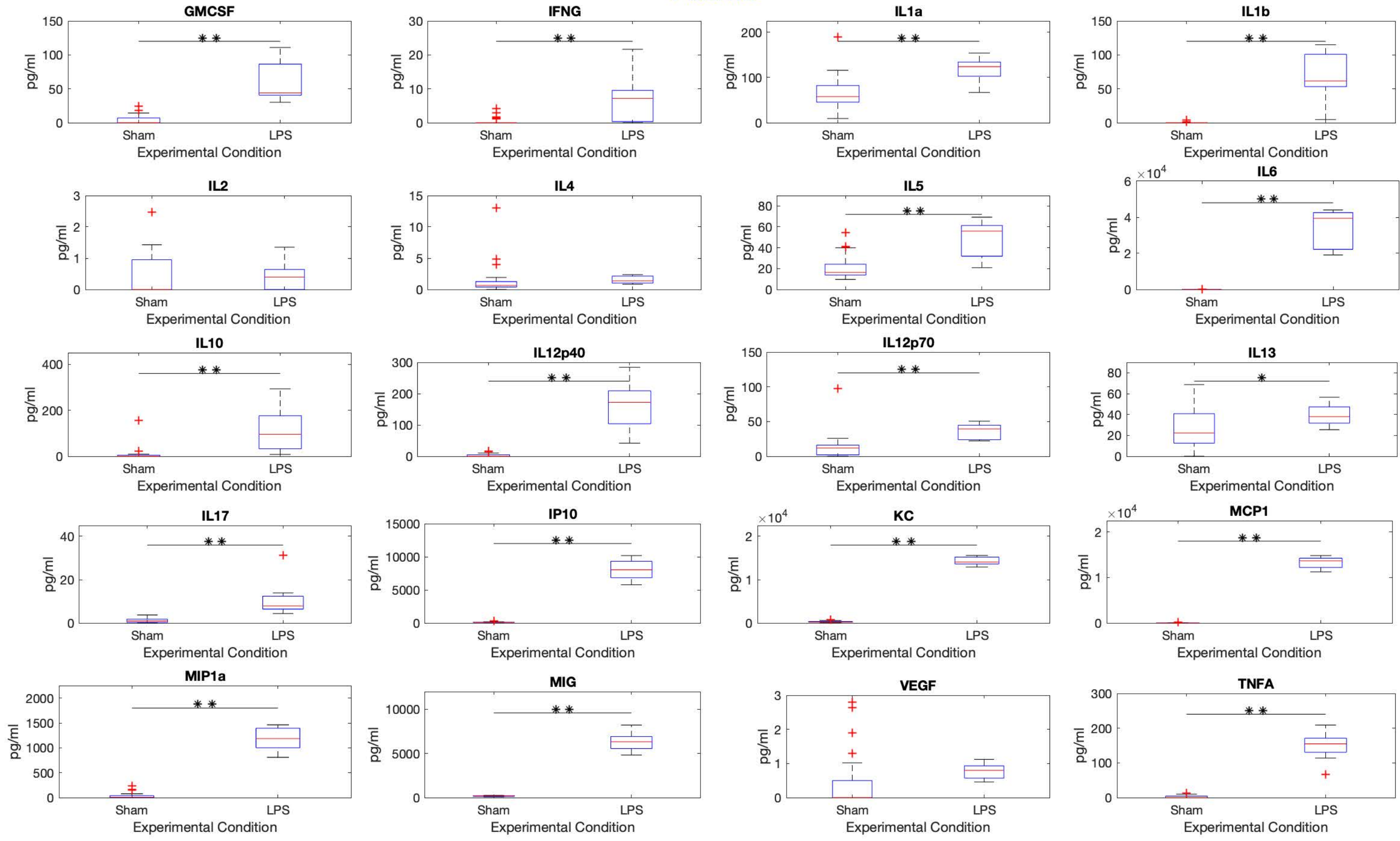

**B**

# Spleen

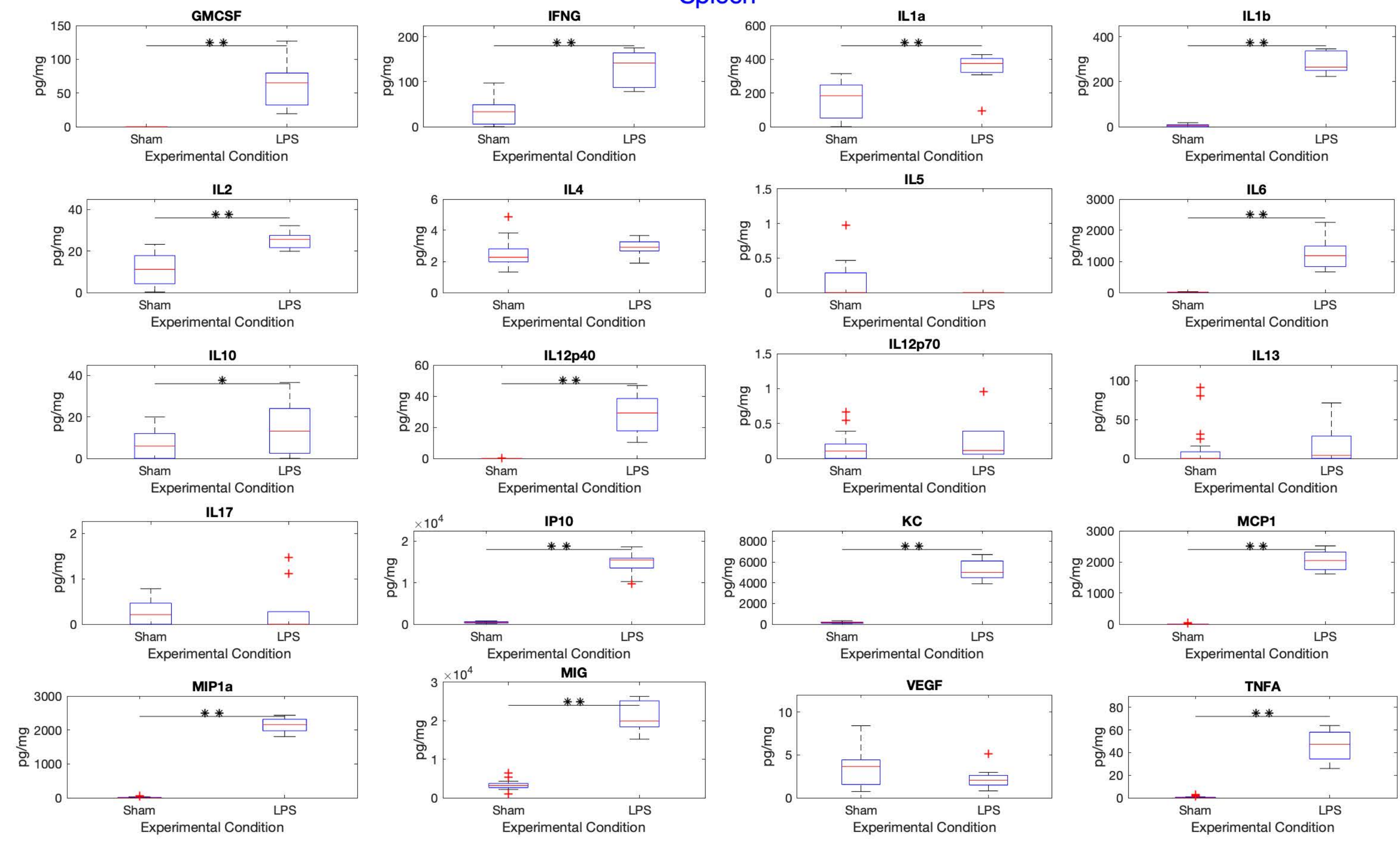

C

Gut

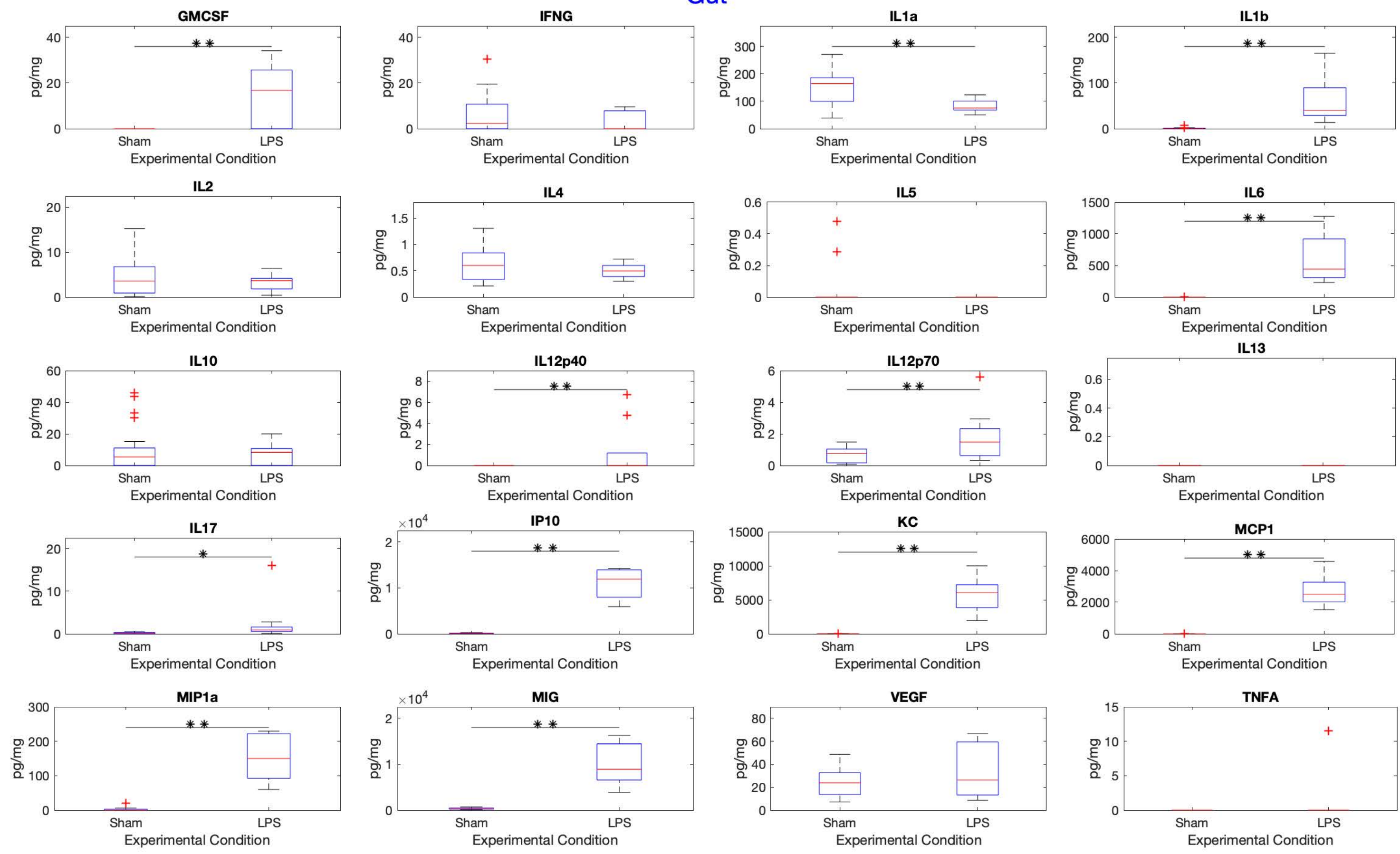

D

Heart

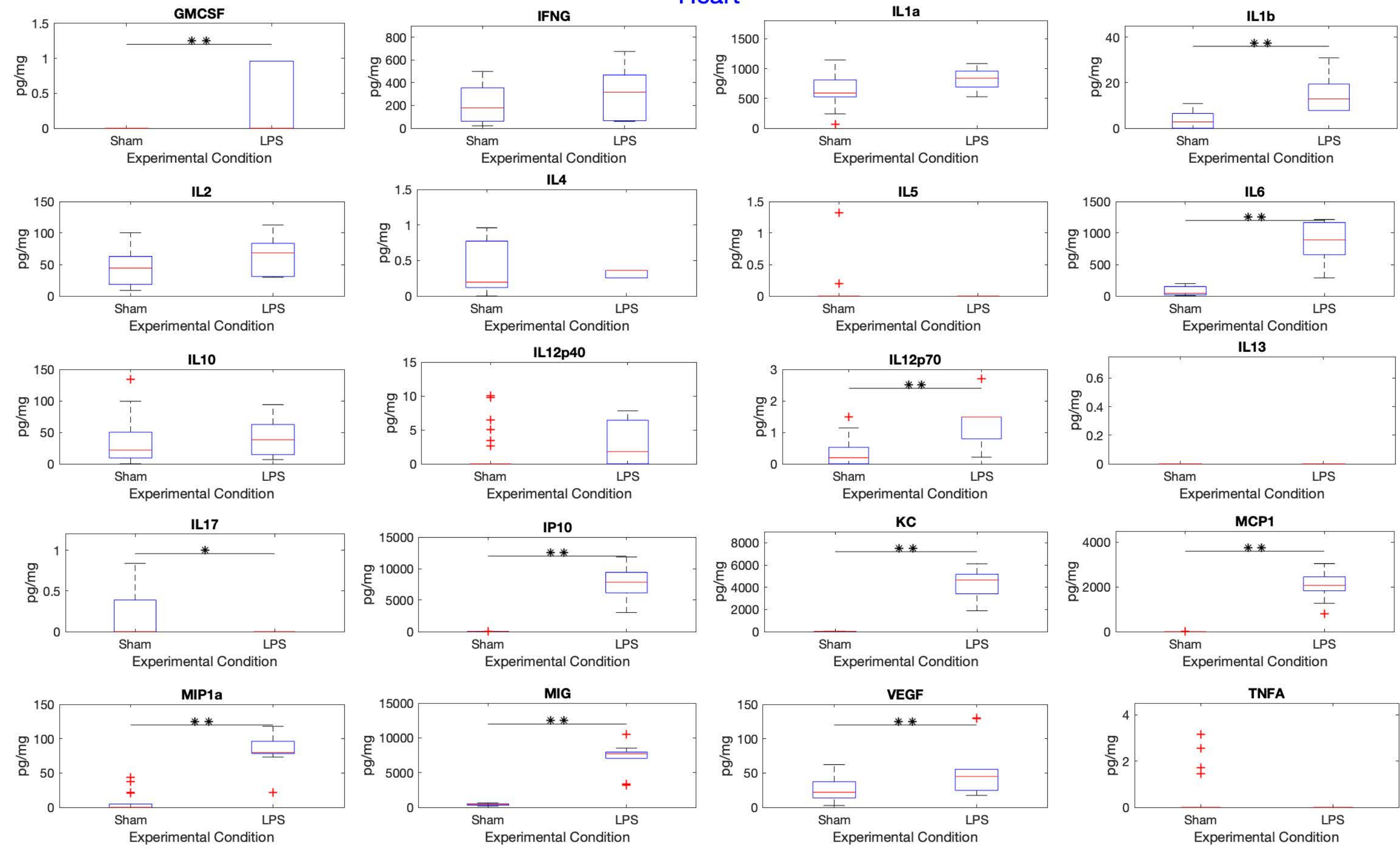

E

Liver

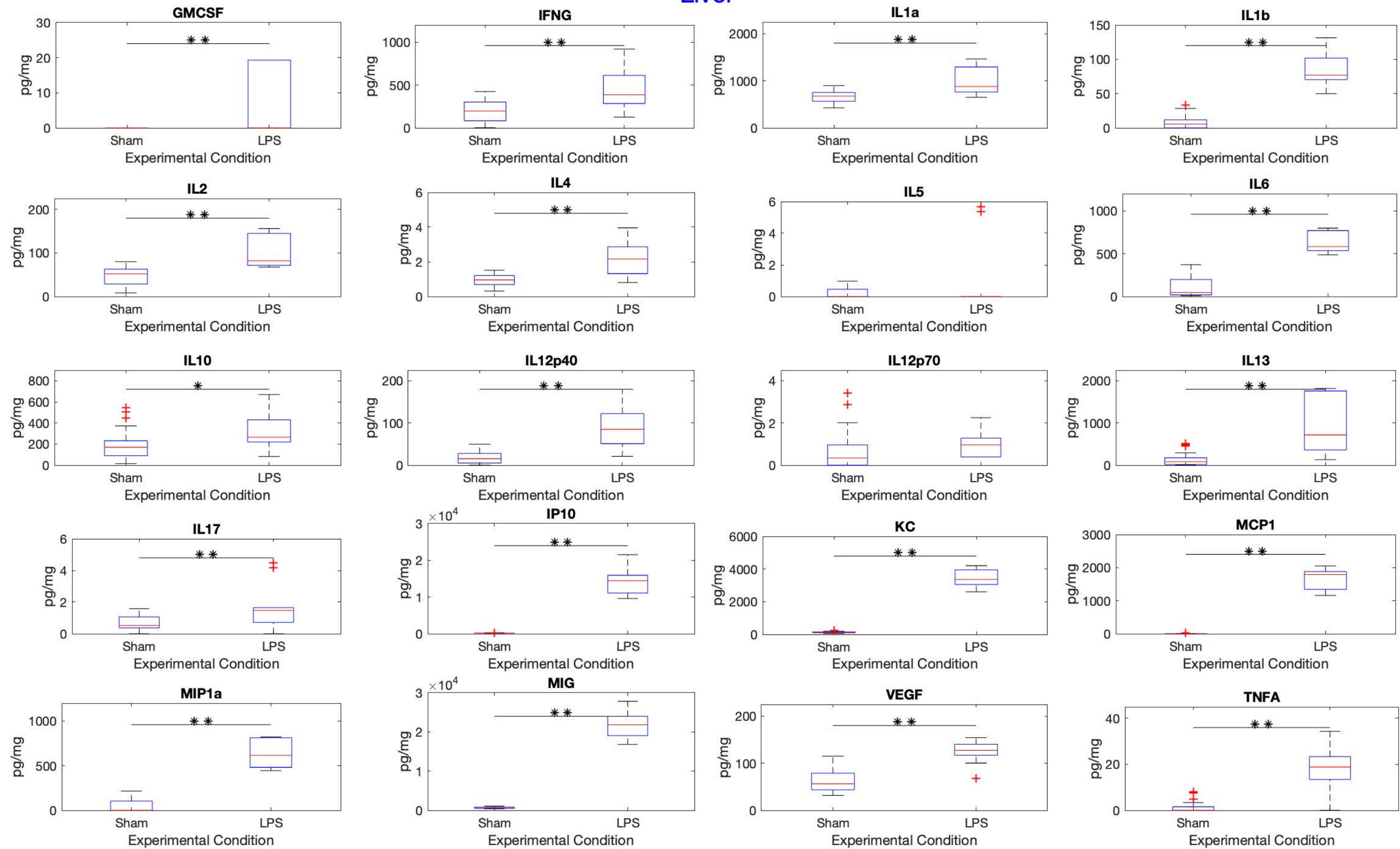

F

Kidney

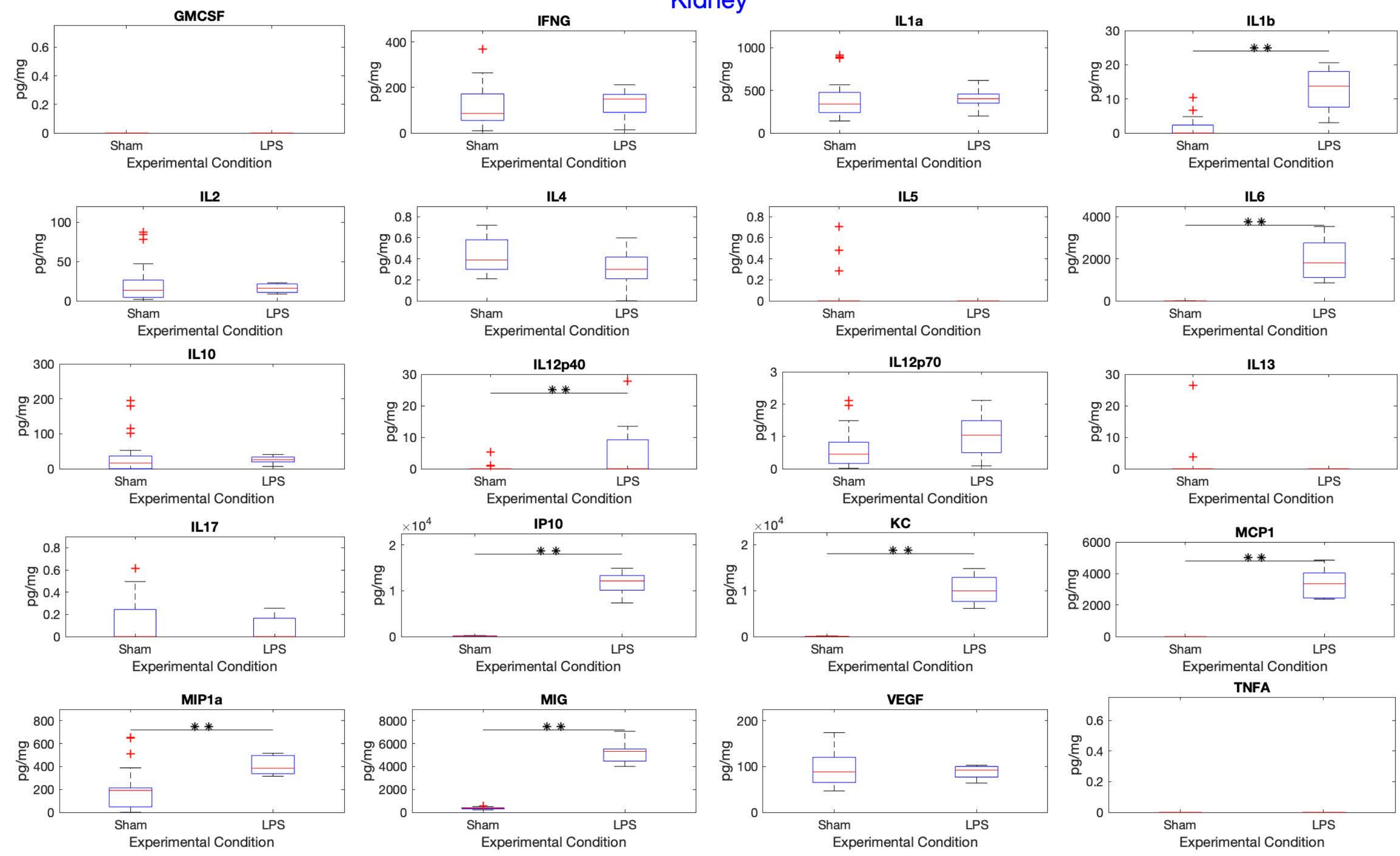

**G****Lung**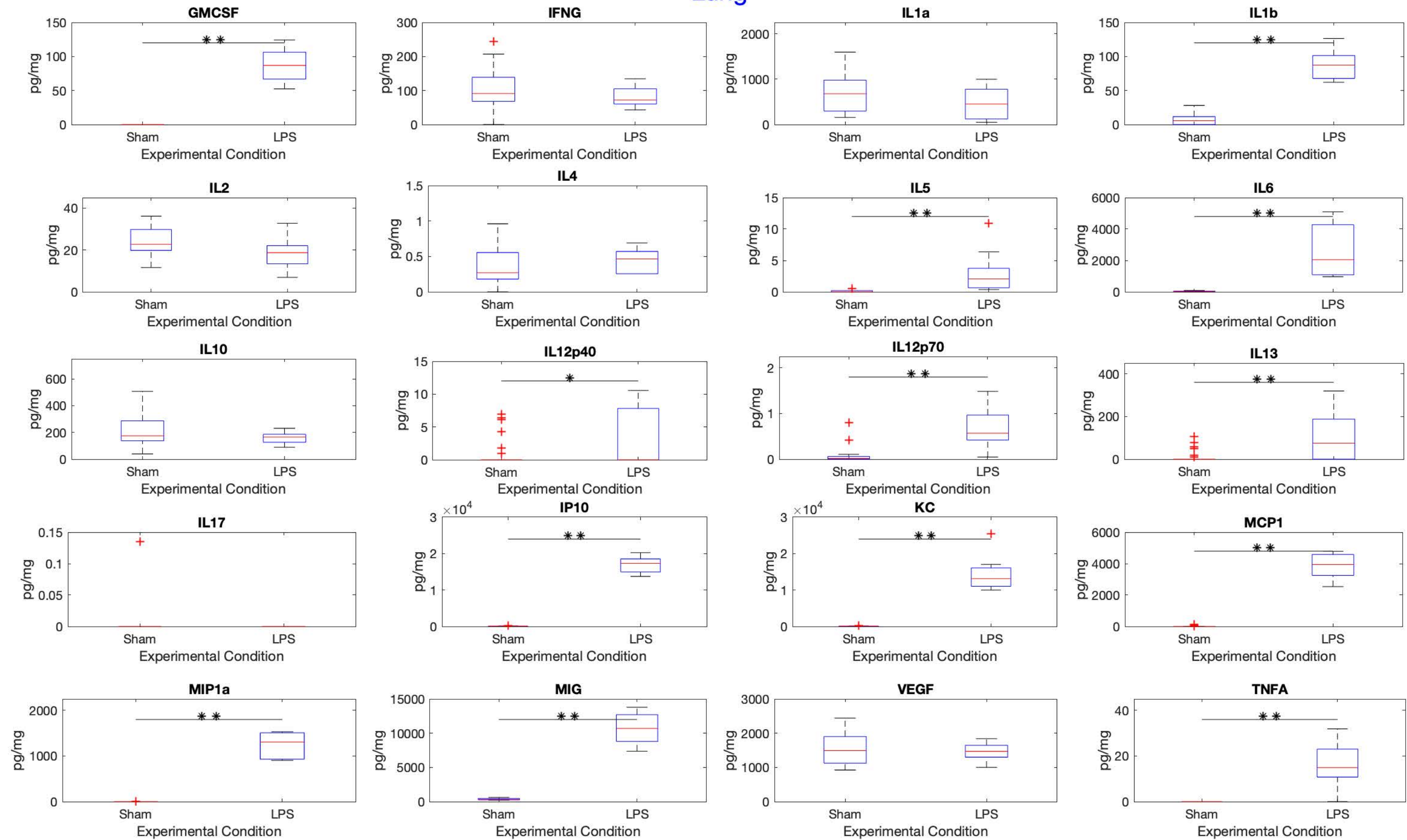

Left Brain

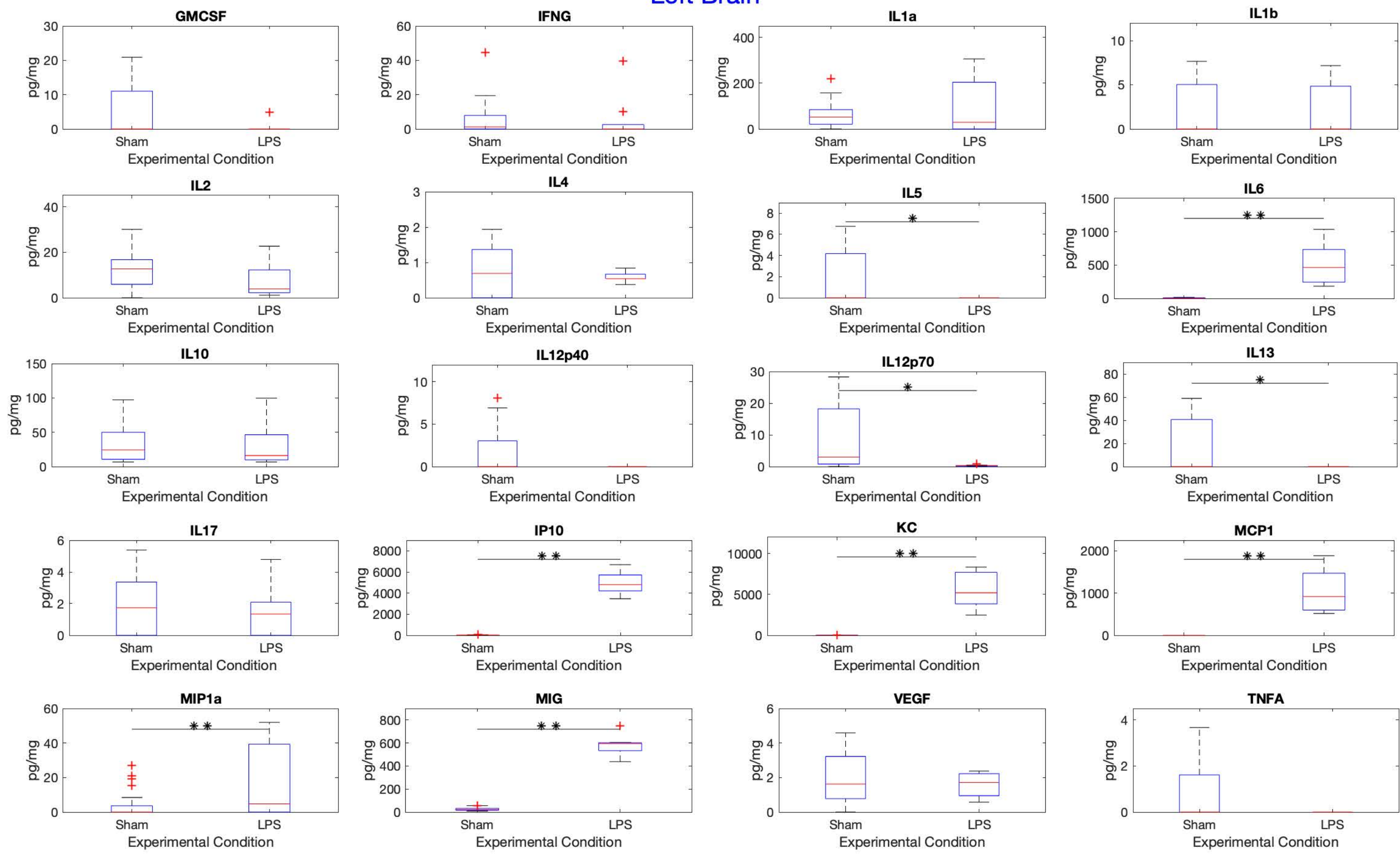

Right Brain

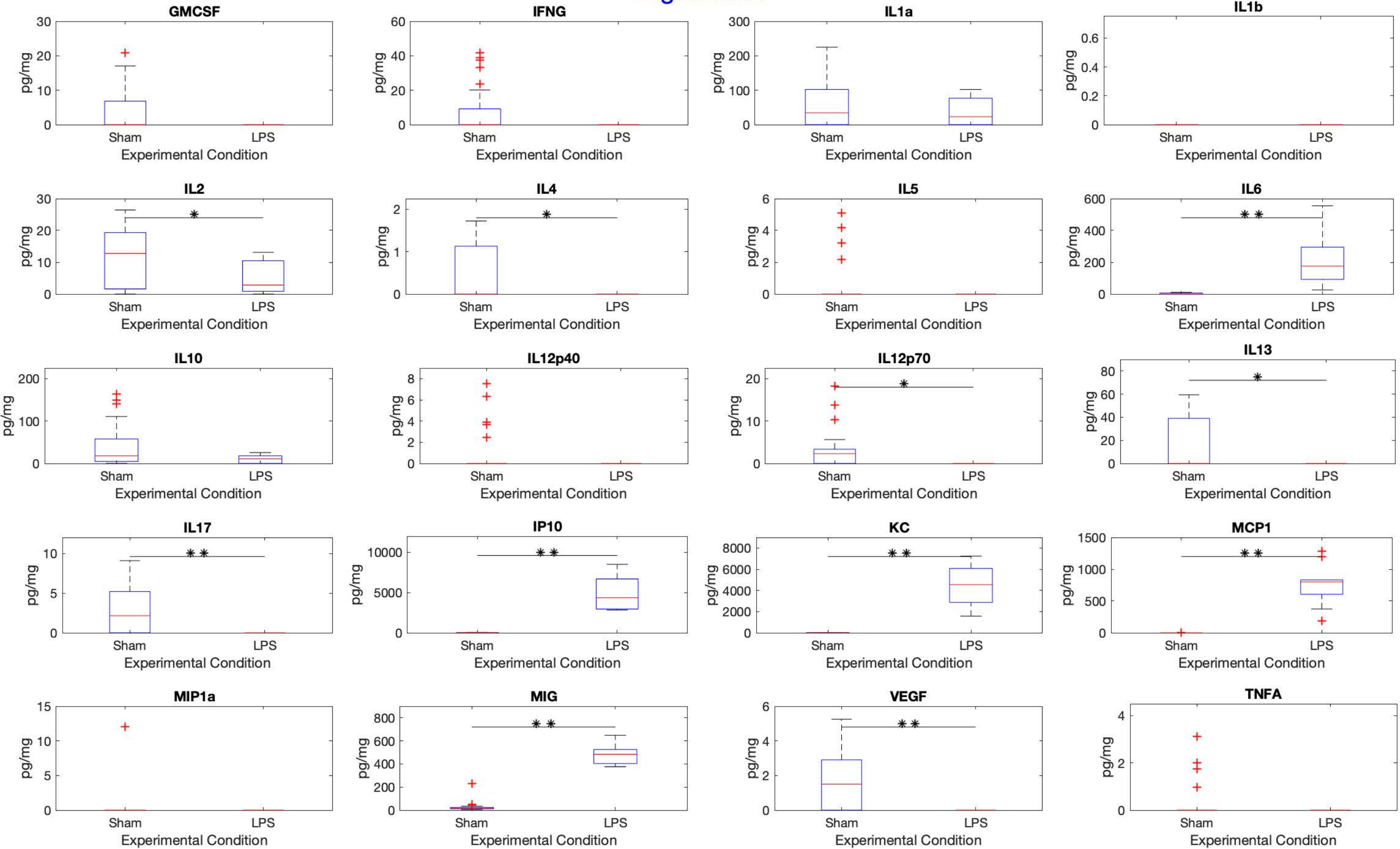

Supplement: Supplementary file 1 [file DataSheet2.PDF]

**A****DyNA Network Complexity**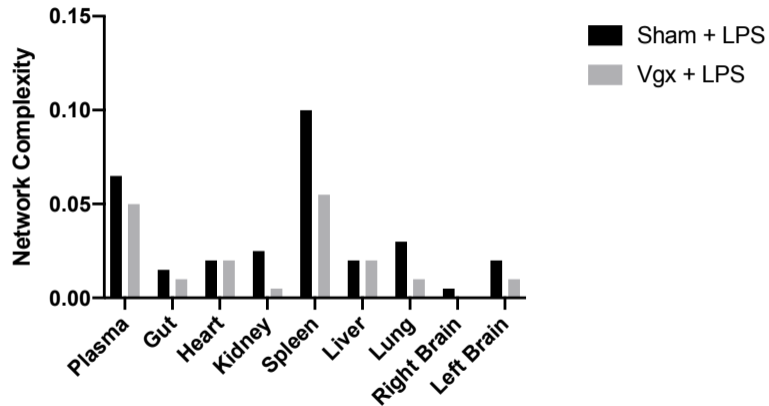**B****DyHyp Network Complexity**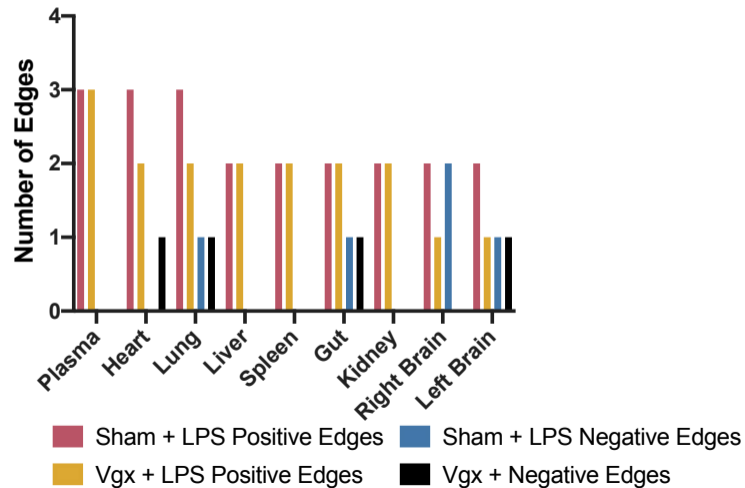

Supplement: Supplementary file 2 [file DataSheet4.PDF]

B

## Sham Surgery

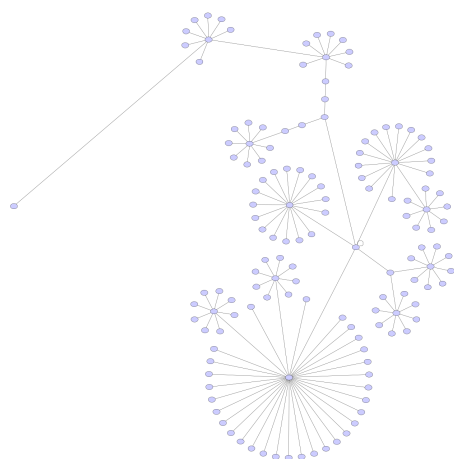

## Vagotomy

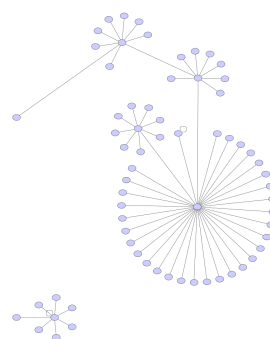

Supplement: Supplementary file 3 [file DataSheet6.PDF]

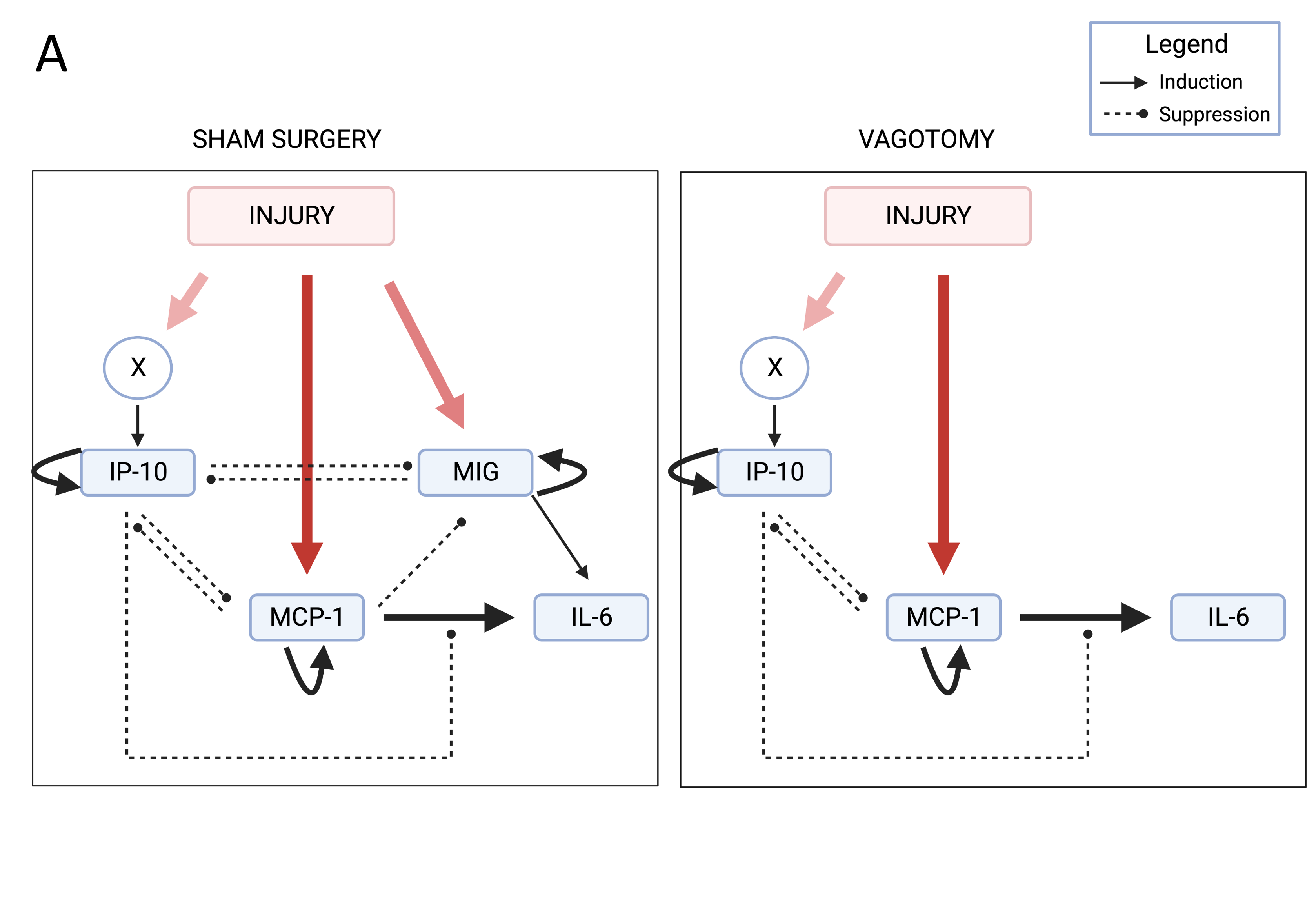

Supplement: Supplementary file 5 [file Image1.TIF]

**A****Plasma**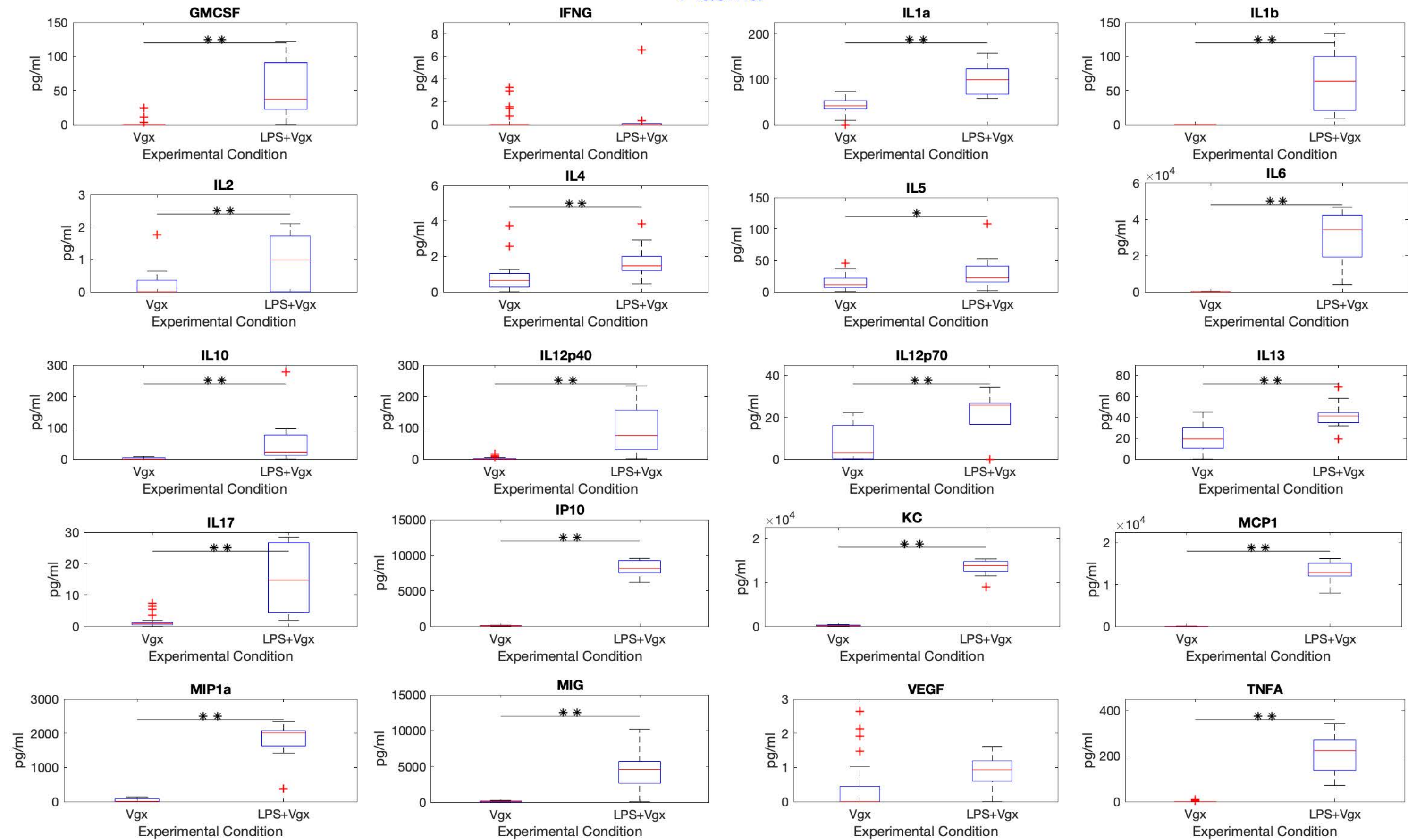

B

Spleen

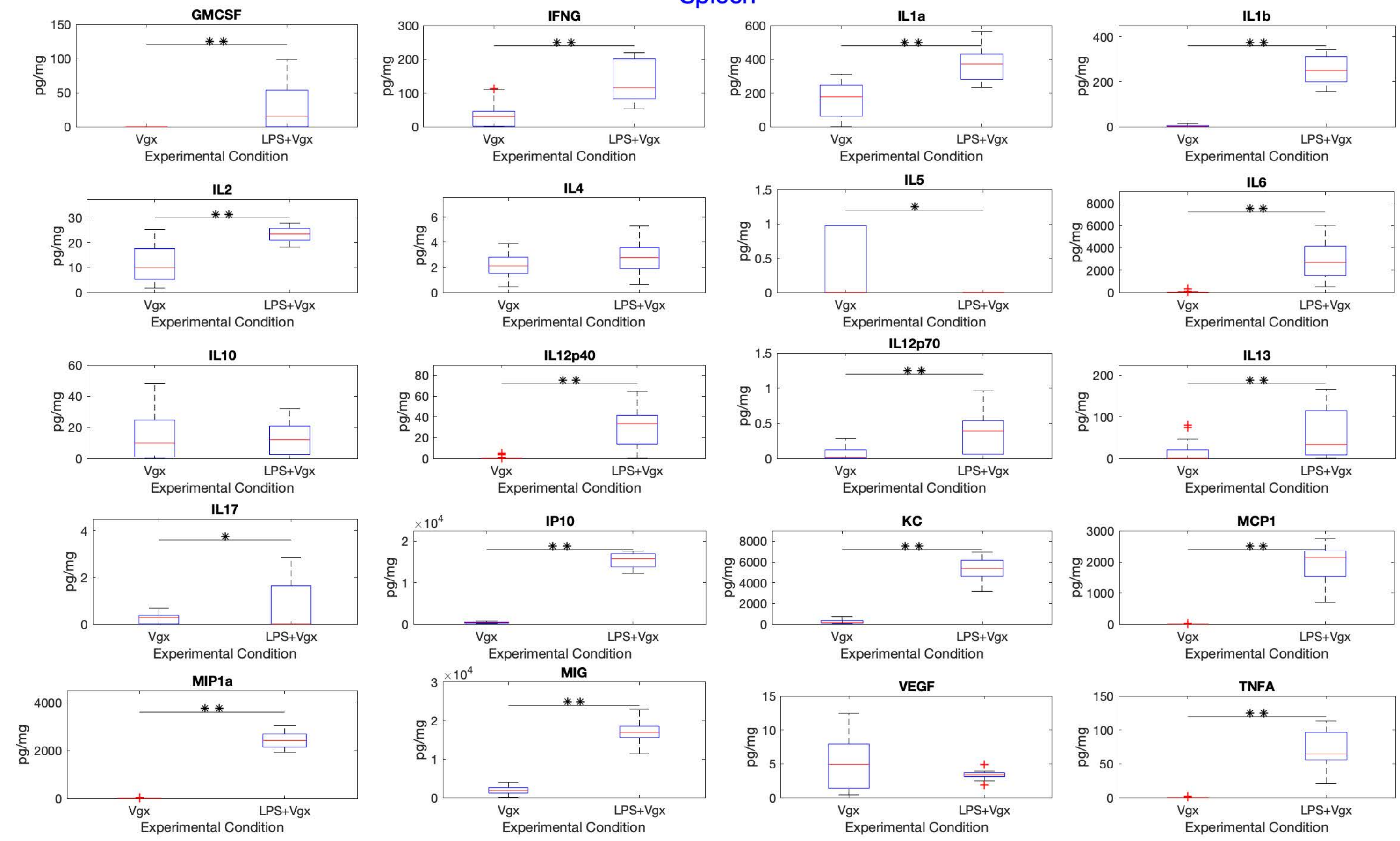

C

Gut

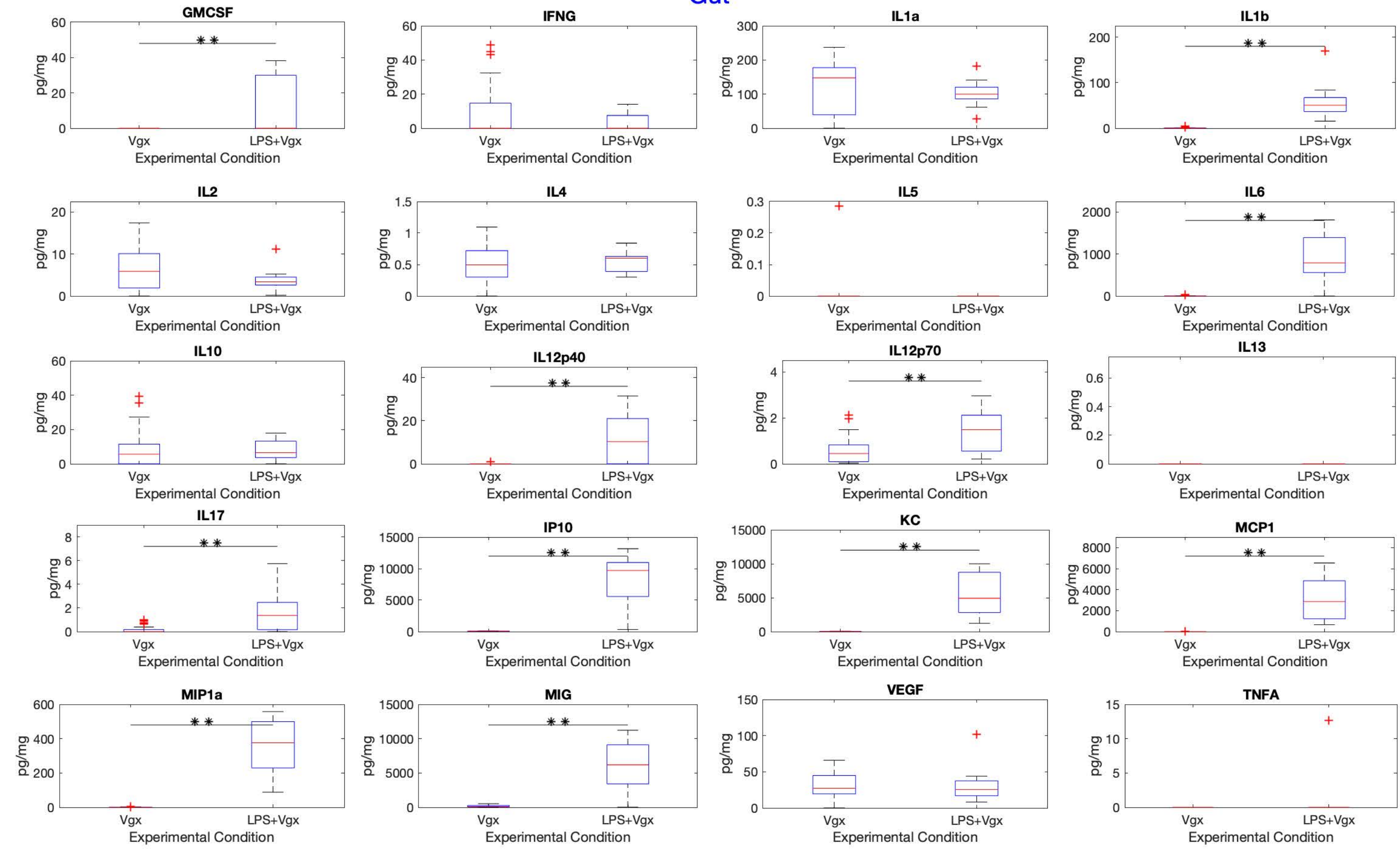

D

## Heart

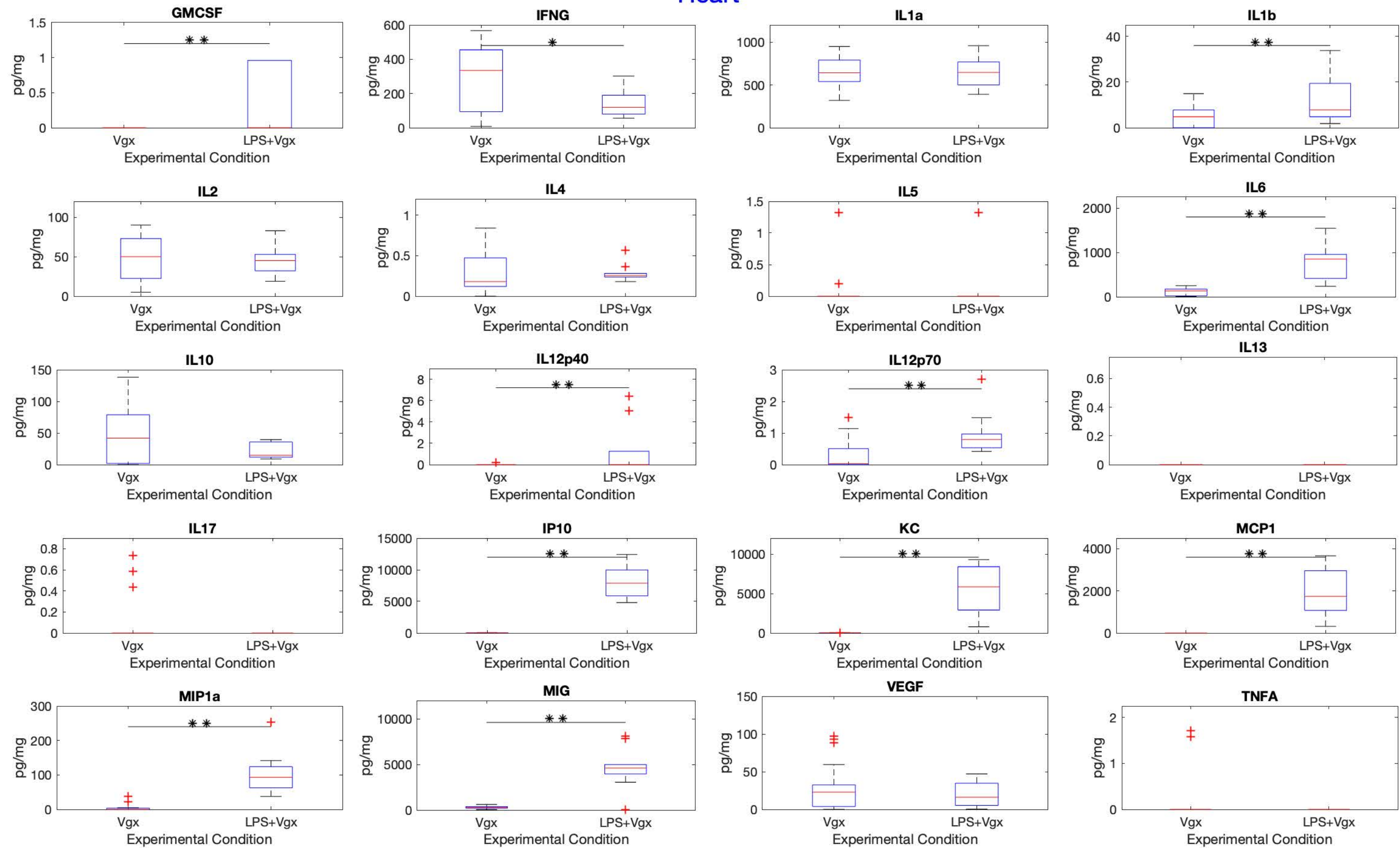

E

Liver

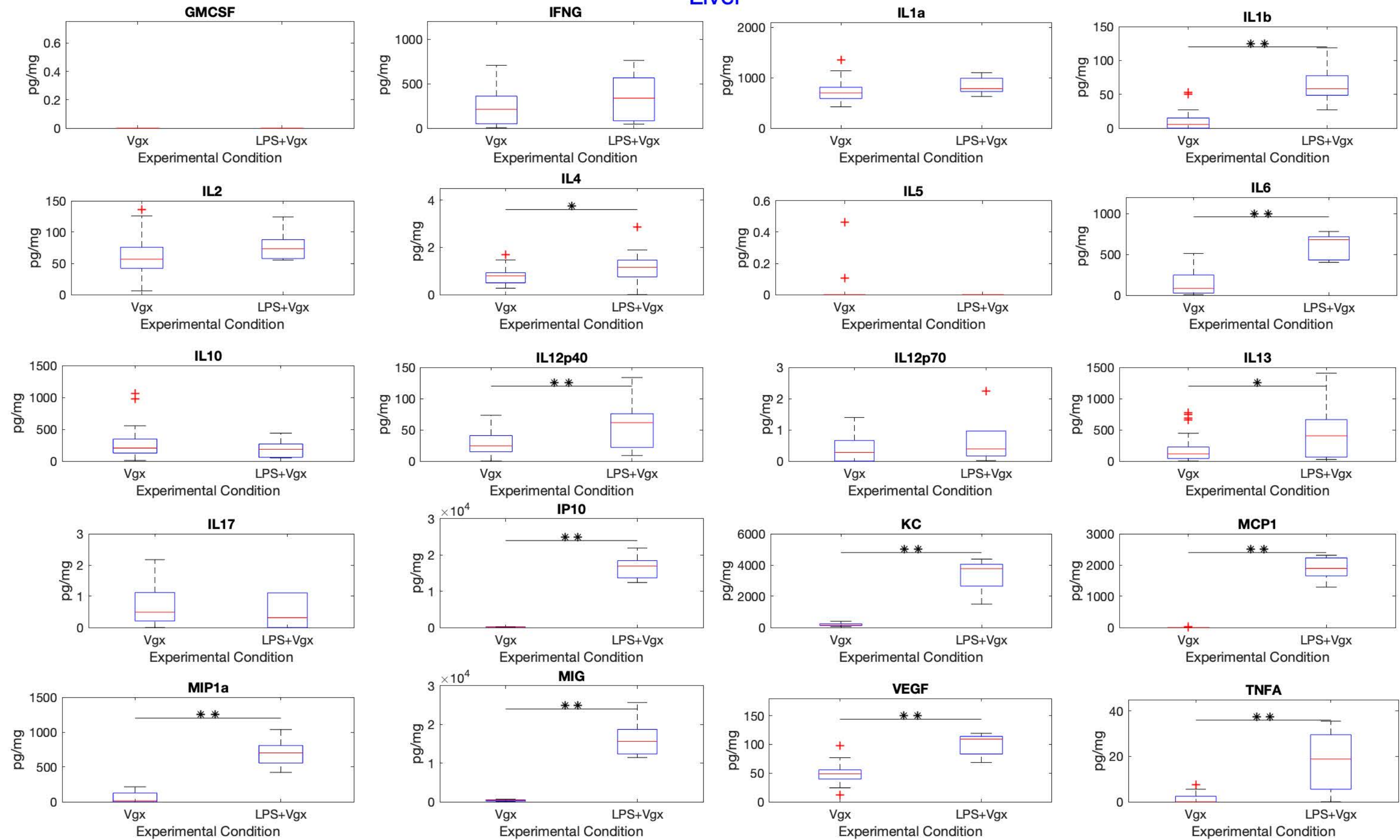

F

Kidney

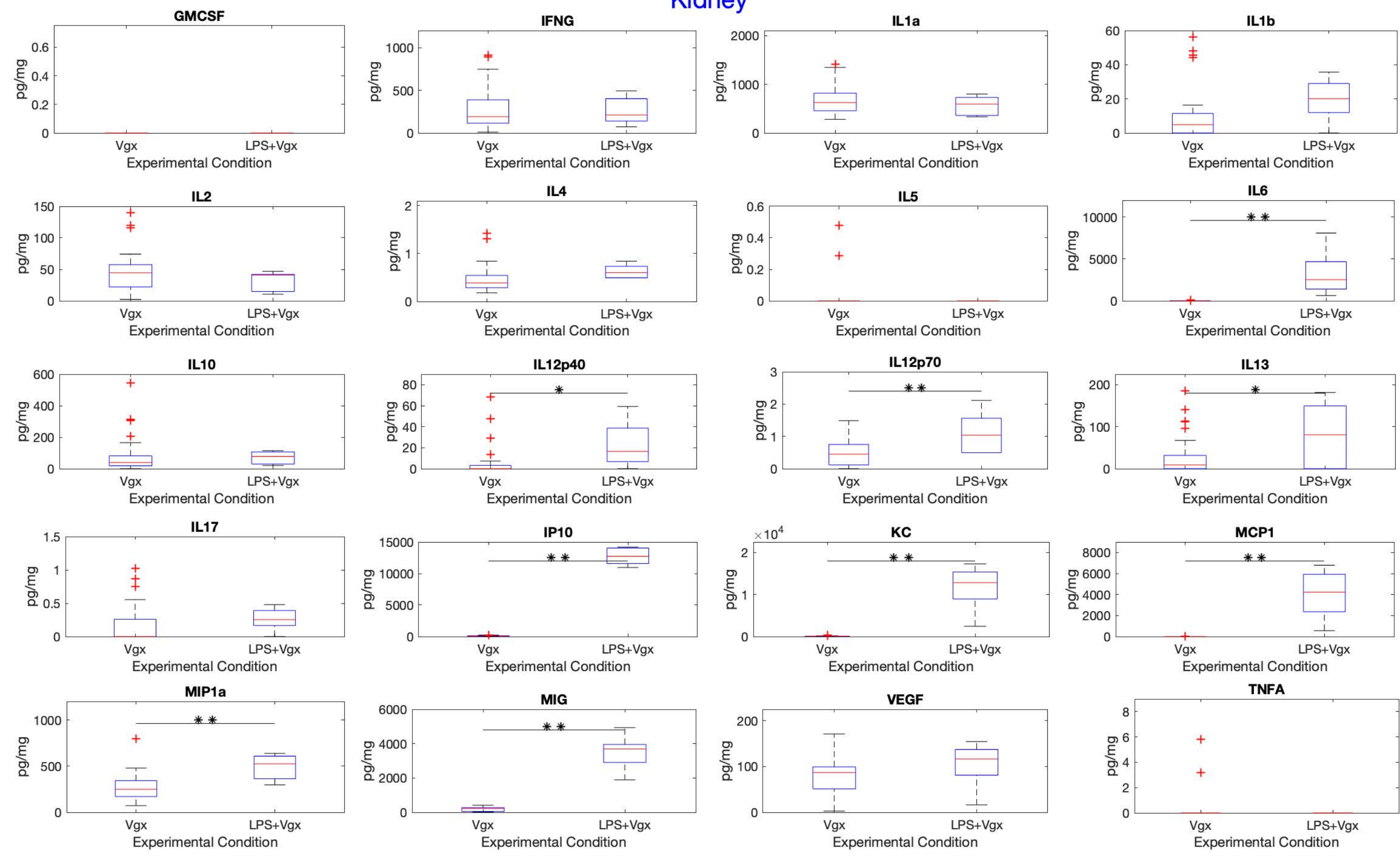

**G****Lung**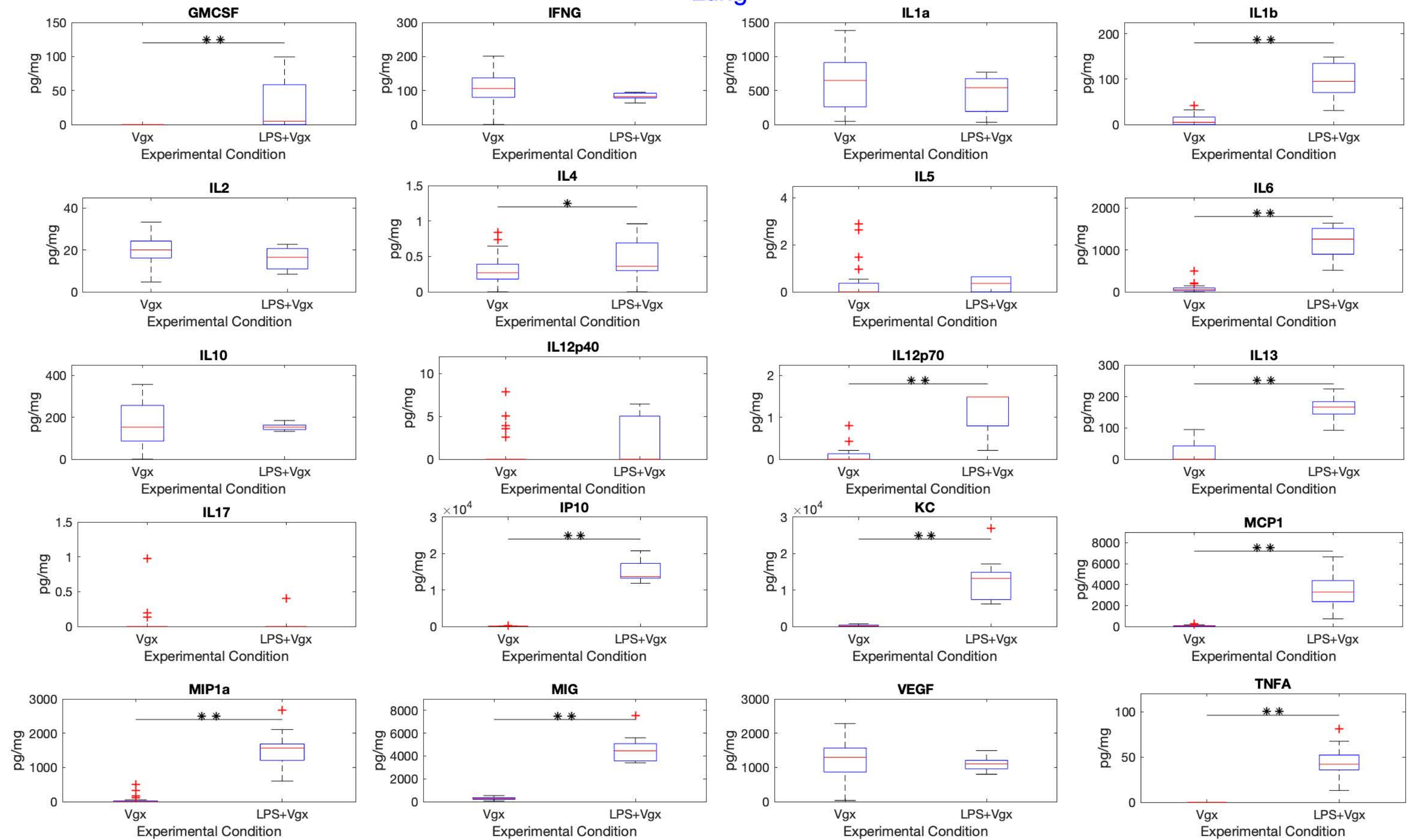

H

Left Brain

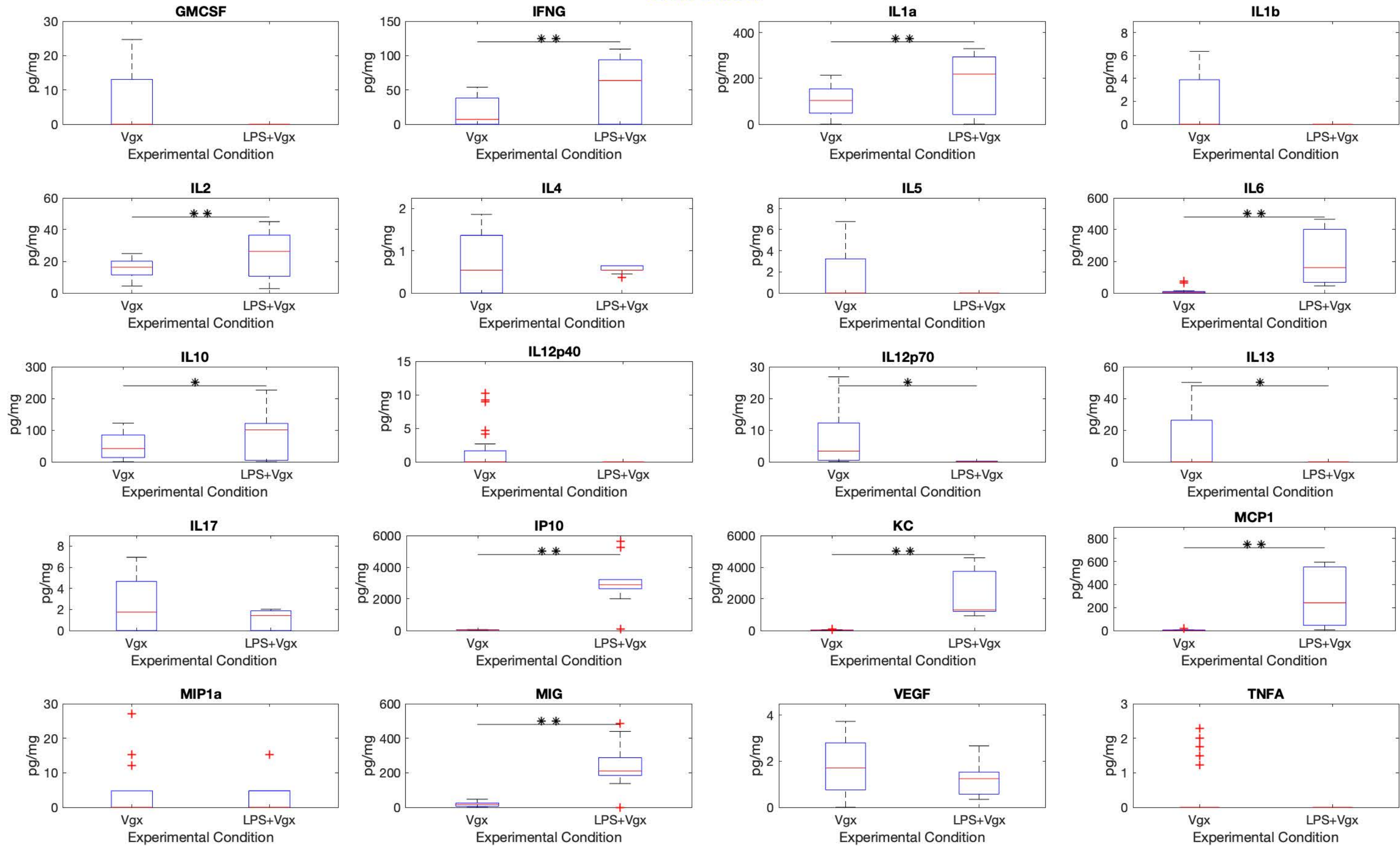

Right Brain

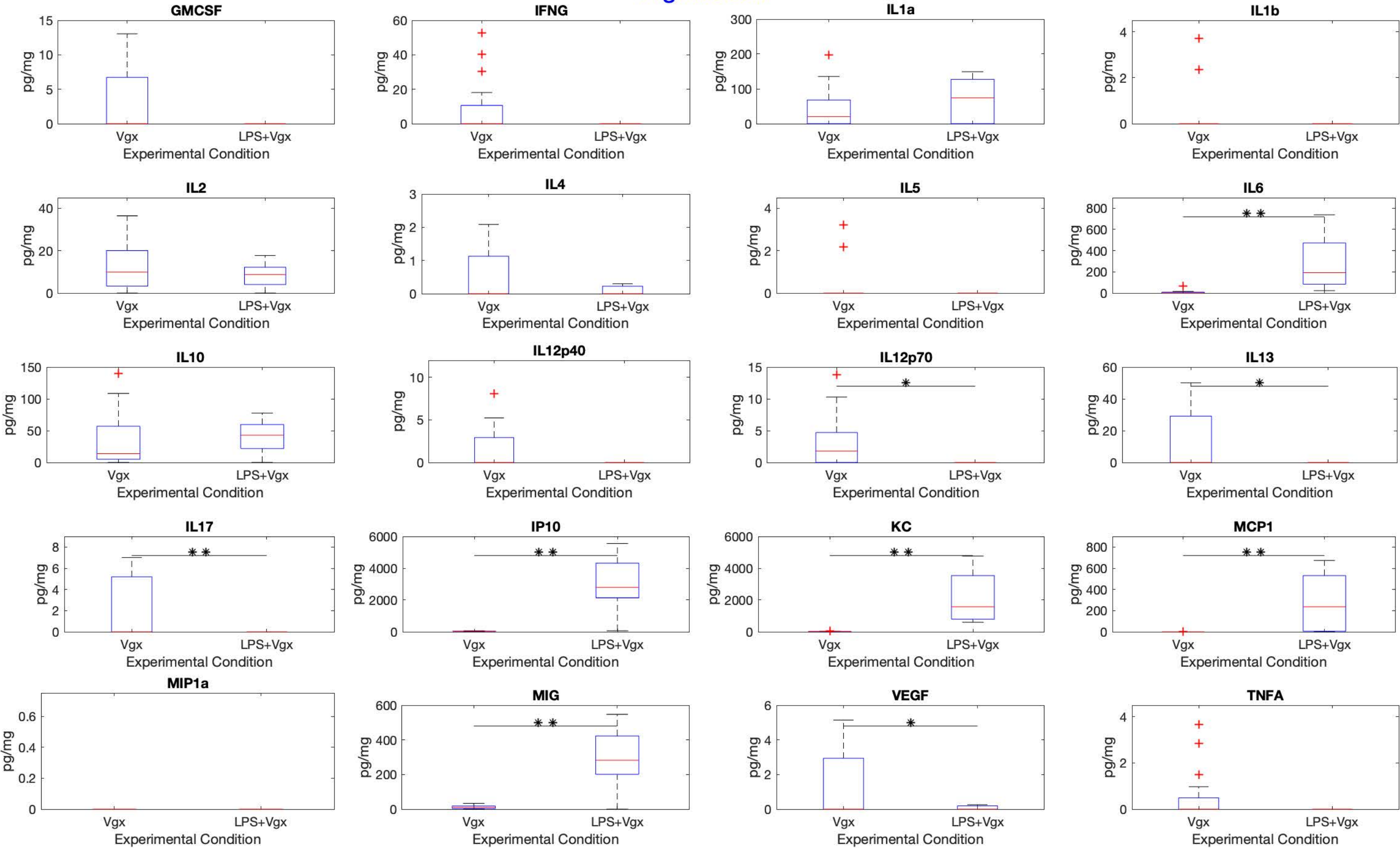

Supplement: Supplementary file 6 [file DataSheet3.PDF]

A

## Plasma

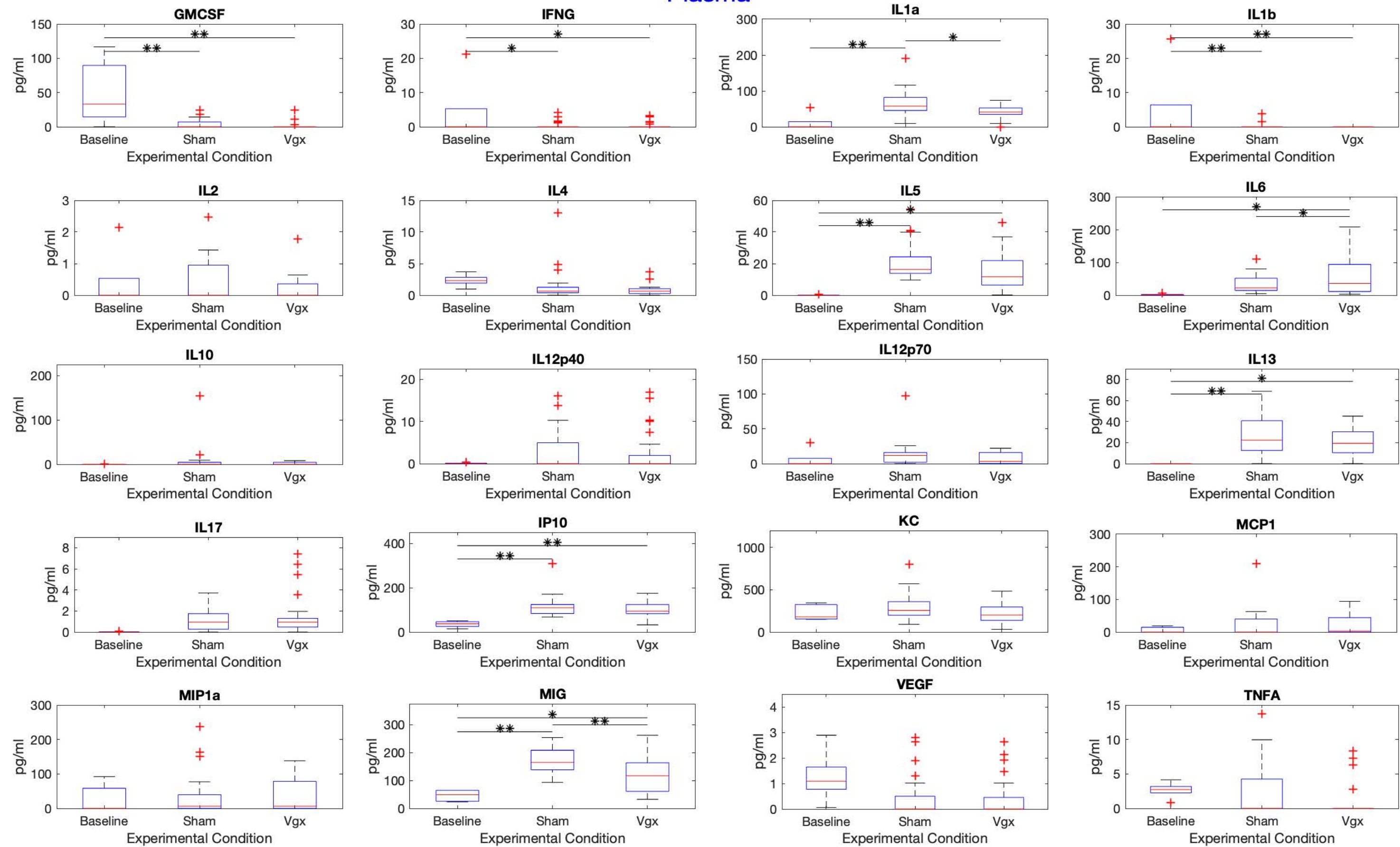

**B****Spleen**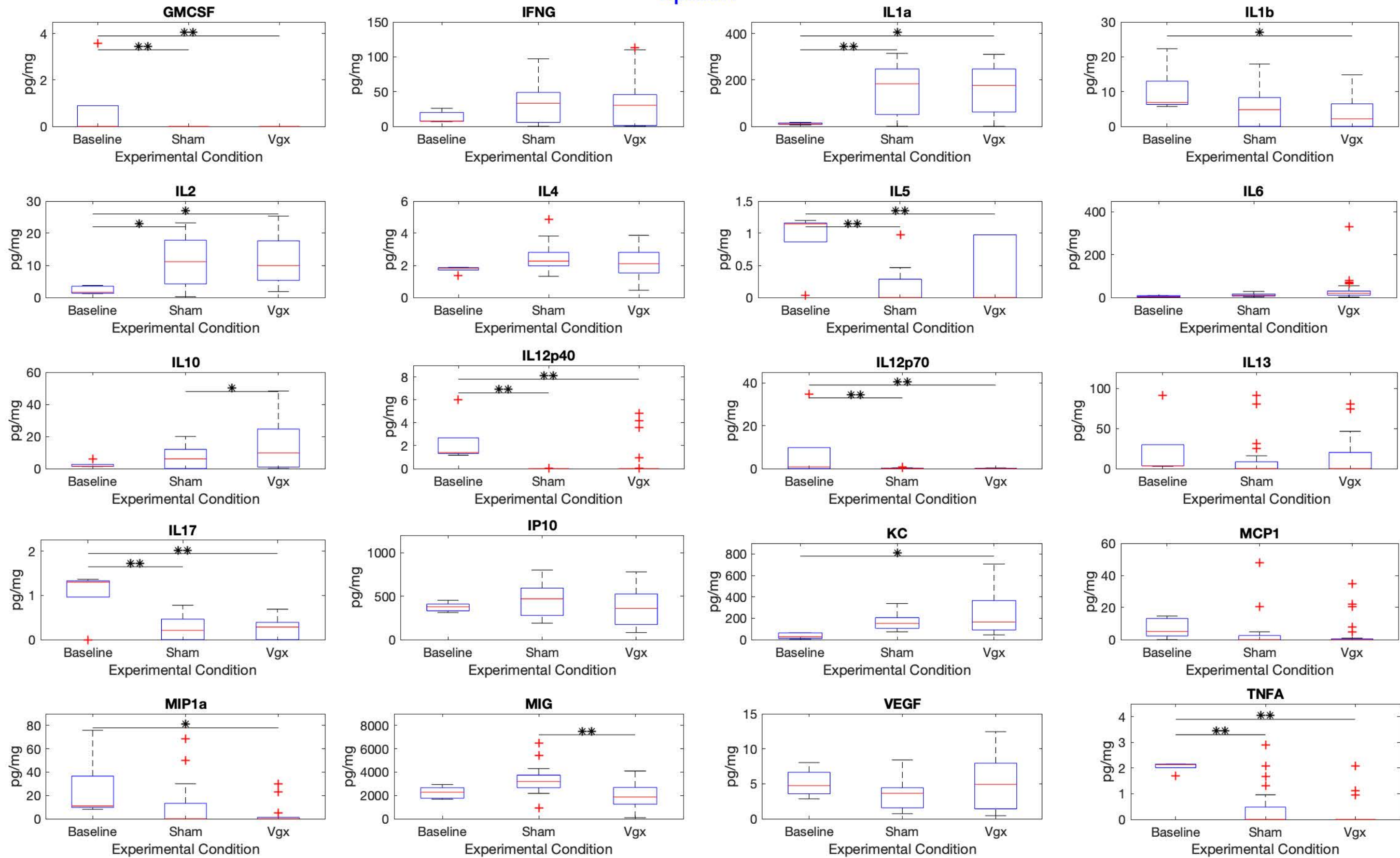

**C****Gut**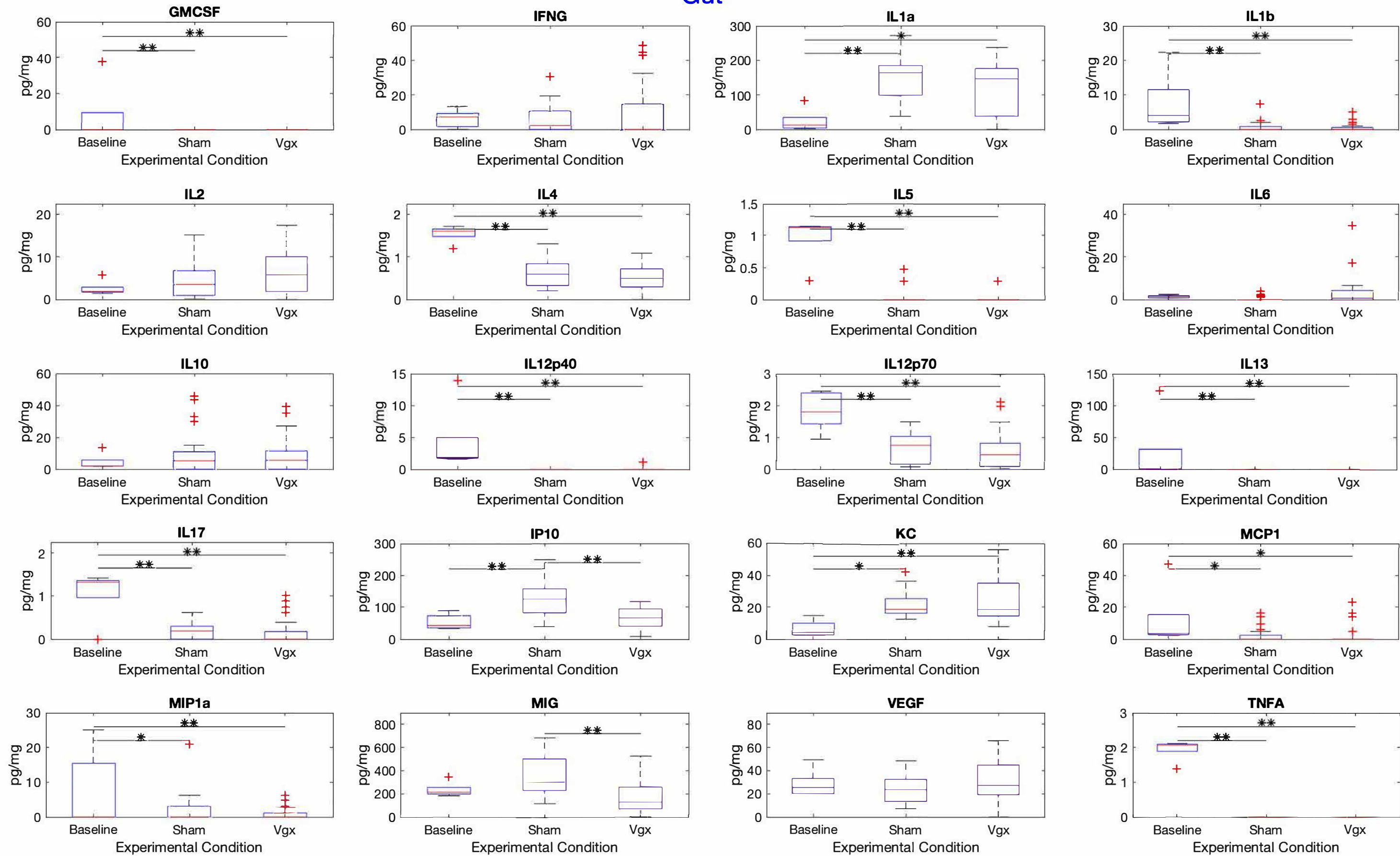

D

# Heart

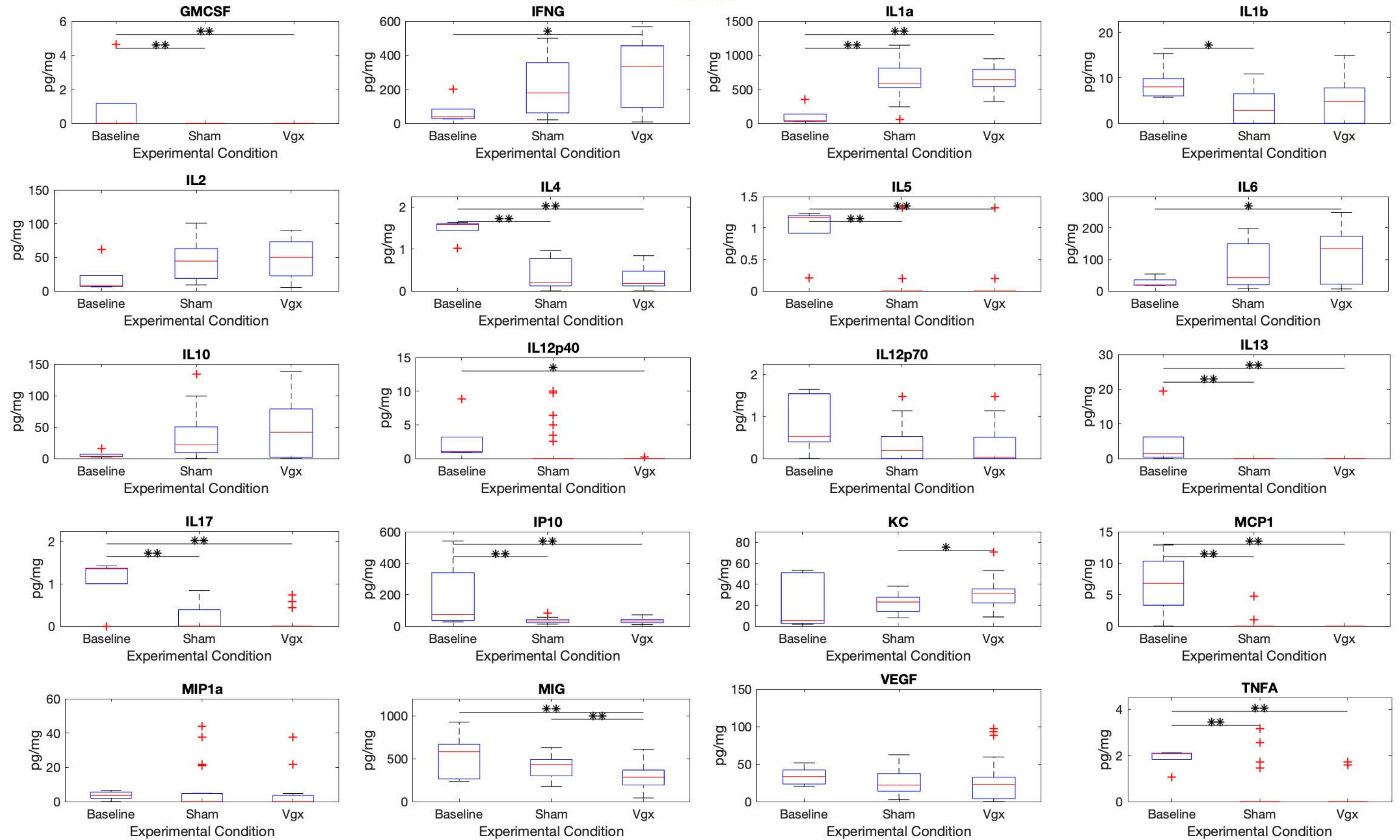

E

Liver

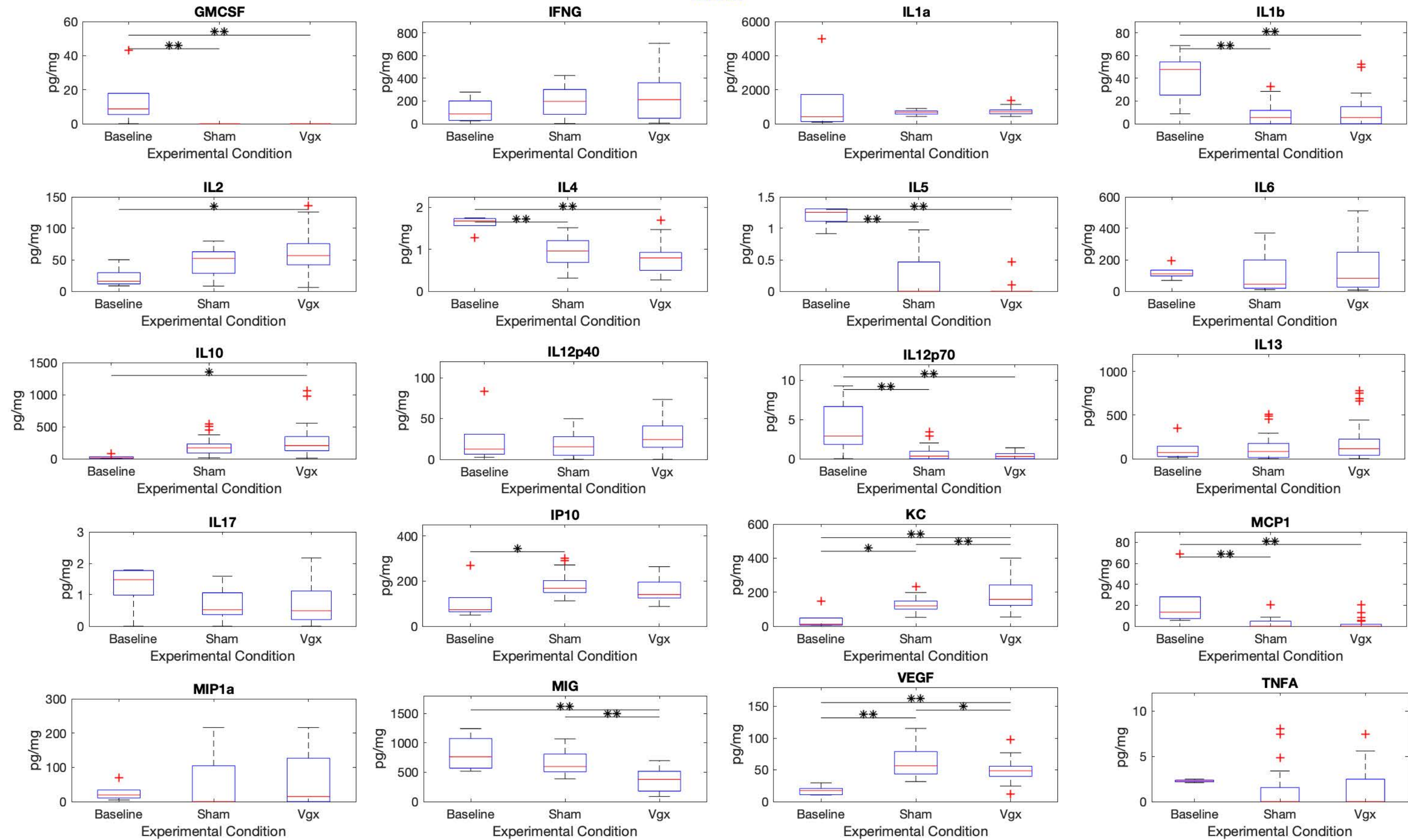

**F****Kidney**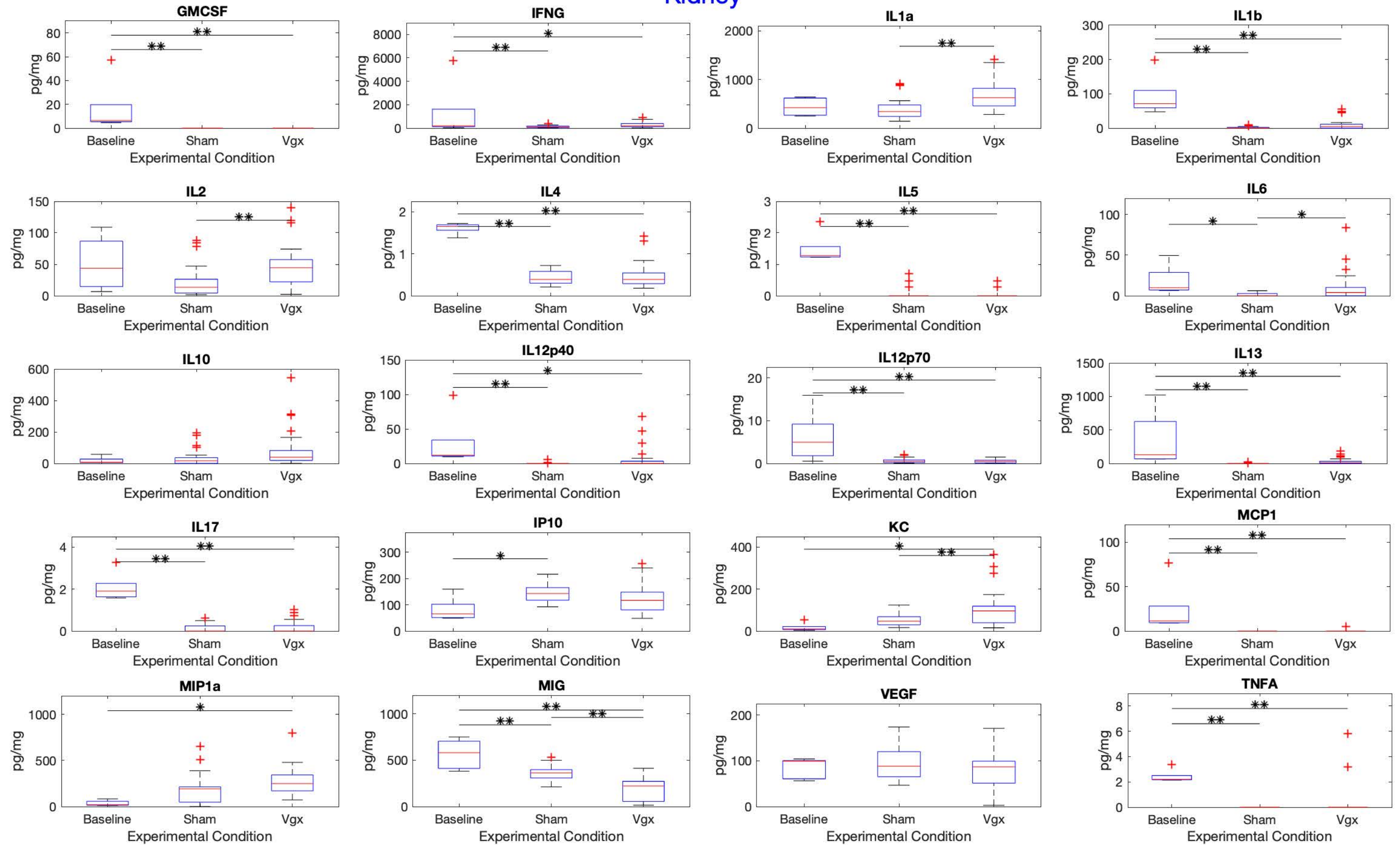

G

## Lung

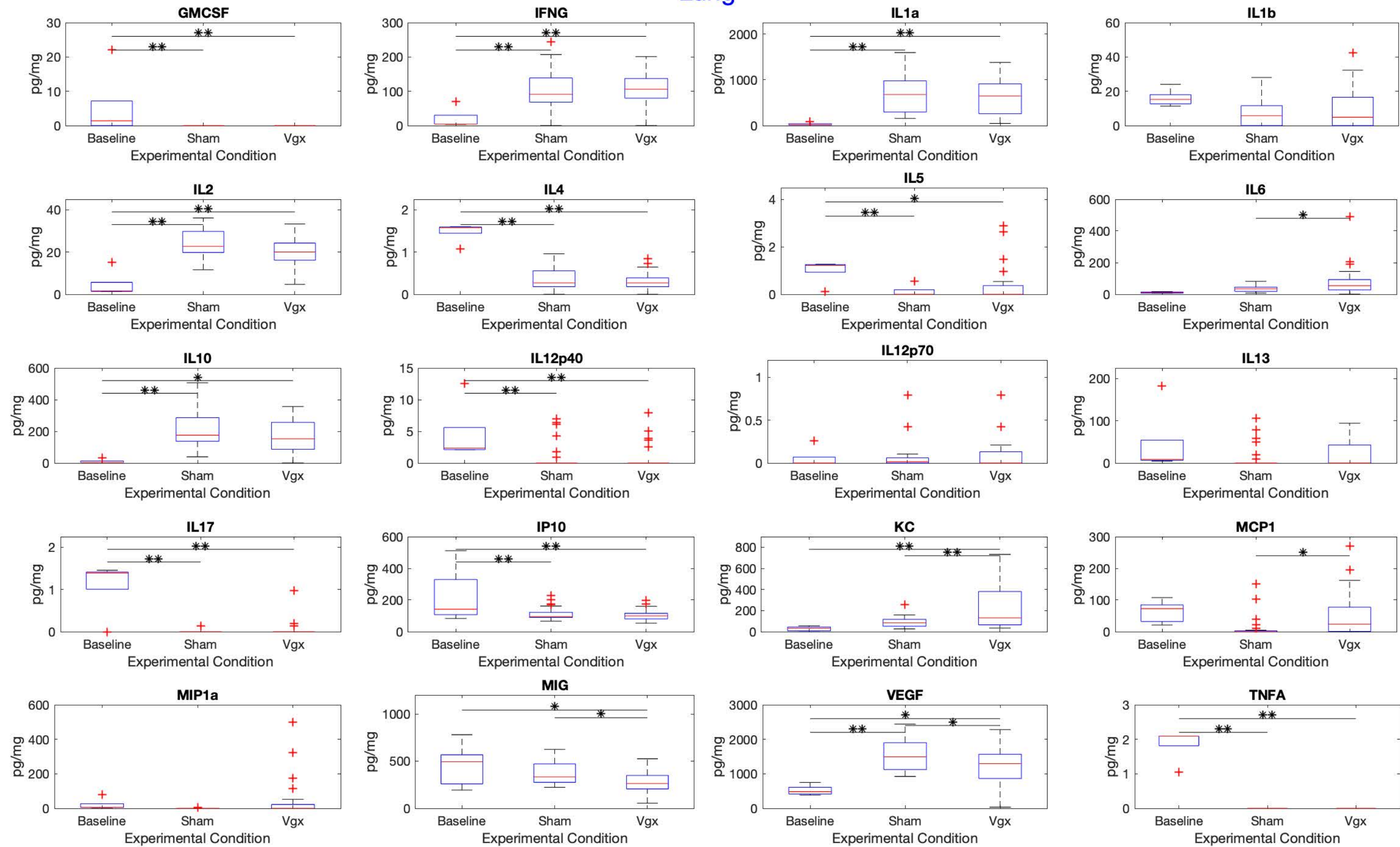

Supplement: Supplementary file 7 [file DataSheet1.PDF]
