# Supplementary material for: Computational inference of chemokine-mediated roles for the vagus nerve in modulating intra- and inter-tissue inflammation
Source: Front Syst Biol. 2024 Feb 15;4:1266279. doi: 10.3389/fsysb.2024.1266279 (PMC12341964; doi:10.3389/fsysb.2024.1266279)
Supplement: Supplementary file 4 [file Table1.DOCX]

**Supplementary Figure Legends**

**Supplementary Figure 1.** Expression of inflammatory mediators in plasma (**A**), spleen (**B**), gut (**C**), heart (**D**), liver (**E**), kidney (**F**), lung (**G**) of mice following either **sham surgery** or **vagotomy** compared to **baseline**. Mediators were measured by Luminex^TM^ technology and results expressed as pg/mg total protein. Mean values are indicated by a red line. The edges of the box-and-whisker plot show data at the 25^th^ and 75^th^ percentile and the whiskers extend to the most extreme data points as described in Materials and Methods.

**Figure supplement 2.** Expression of inflammatory mediators in plasma (**A**), spleen (**B**), gut (**C**), heart (**D**), liver (**E**), kidney (**F**), lung (**G**), left brain (**H**) and right brain (**I**) of mice following **LPS challenge** compared to **baseline**. Mediators were measured by Luminex^TM^ technology and results are expressed as pg/mg total protein (mean ± S.E.M). The edges of the box-and-whisker plot show data at the 25^th^ and 75^th^ percentile and the whiskers extend to the most extreme data points as described in Materials and Methods.

**Figure supplement 3.** Expression of inflammatory mediators in plasma (**A**), spleen (**B**), gut (**C**), heart (**D**), liver (**E**), kidney (**F**), lung (**G**), left brain (**H**) and right brain (**I**) of **vagotomized mice** with and without **LPS challenge**. Mediators were measured by Luminex^TM^ technology and results are expressed as pg/mg total protein (mean ± S.E.M). The edges of the box-and-whisker plot show data at the 25^th^ and 75^th^ percentile and the whiskers extend to the most extreme data points as described in Materials and Methods.

**Figure supplement 4. Network complexity** in plasma, spleen, gut, heart, liver, kidney, lung, left brain and right brain of **sham** and **vagotomized mice** after **LPS challenge**. Mediators were measured by Luminex^TM^ technology and DyNA (**A**) and DyHyp (**B**) was performed as described in Materials and Methods.

**Figure supplement 5. Boolean network** showing a transition state diagram with a lower degree of network complexity in the model of vagotomy (**B**) compared to sham surgery (**A**).

**Table supplement 1. Hallmarks of the systems biology-derived model** showing the effects of vagotomy on regulating inter- and intra-tissue inflammatory networks.
