# Supplementary material for: Computational inference of chemokine-mediated roles for the vagus nerve in modulating intra- and inter-tissue inflammation
Source: Front Syst Biol. 2024 Feb 15;4:1266279. doi: 10.3389/fsysb.2024.1266279 (PMC12341964; doi:10.3389/fsysb.2024.1266279)
Supplement: Supplementary file 8 [file DataSheet5.PDF]

Sham Surgery		
Intra-tissue Networks	Inter-tissue Networks	Rate-of-Change
Plasma:<br>IFNγ ←→ IL-1β<br><br>Kidney and Lung:<br>TNFα ←→ IL-17A	Positive Edges:<br>MIG: plasma, spleen<br><br>Negative Edges:<br>IFNγ: kidney, gut<br>MCP1: lung, gut<br>MIG: kidney, lung, liver, heart<br>MIP-1α: spleen	$v_{\text{IP-10, Spleen}} > 0$<br>$v_{\text{MIG, Spleen}} > 0$<br>$v_{\text{IL-6, Heart}} \propto v_{\text{MCP-1, Heart}}$

Vagotomy		
Intra-tissue Networks	Inter-tissue Networks	Rate-of-Change
Plasma:<br>IFNγ ←→ IL-1β<br><br>Heart:<br>IFNγ ←→ IL-6	Positive Edges:<br>MIG: plasma<br>IP-10: plasma<br><br>Negative Edges:<br>MIG: kidney, lung, liver, spleen, gut, heart	$v_{\text{IP-10, Spleen}} < 0$<br>$v_{\text{MIG, Spleen}} < 0$<br>$v_{\text{IL-6, Heart}} > v_{\text{MCP-1, Heart}}$

Sham Surgery + LPS		
Intra-tissue Networks	Inter-tissue Networks	Rate-of-Change
Plasma, kidney, spleen, heart:<br>IL-6 ←→ MCP-1<br><br>Plasma: MIG ←→ IP-10, MIP-1α, MCP-1, KC<br><br>Spleen: IL-6 ←→ MCP-1, KC, IL-12p40	Positive Edges:<br>IL-6, MCP-1: plasma<br>KC: brain, kidney, lung, heart<br>MIG: spleen, lung, liver, heart, gut<br><br>Negative Edges:<br>IL-1α: brain, lung, gut	$v_{\text{IFN}\gamma\text{, Heart}} > 0$<br>$v_{\text{MIG, Spleen}} < v_{\text{MIG, Liver}}$<br>$v_{\text{IL-6}} \propto v_{\text{MCP-1}}$

Vagotomy + LPS		
Intra-tissue Networks	Inter-tissue Networks	Rate-of-Change
Plasma:<br>IL-6 ←→ TNFα<br><br>Heart:<br>IFNγ ←→ IL-6	Positive Edges:<br>IL-6, MCP-1: plasma<br>KC: kidney, lung, heart<br>MIG: spleen, liver, gut<br><br>Negative Edges:<br>IL-1α: gut<br>IFNγ: heart	$v_{\text{IFN}\gamma\text{, Heart}} < 0$<br>$v_{\text{MIG, Spleen}} = v_{\text{MIG, Liver}}$<br>$v_{\text{IL-6}} \propto v_{\text{MCP-1}}$
